# Potent Cross‐neutralizing Antibodies Reveal Vulnerabilities of Henipavirus Fusion Glycoprotein

**DOI:** 10.1002/advs.202501996

**Published:** 2025-04-29

**Authors:** Yi Ren, Pengfei Fan, Xinghai Zhang, Ting Fang, Zhengshan Chen, Yanfeng Yao, Xiangyang Chi, Guanying Zhang, Xiaofan Zhao, Bingjie Sun, Fangxu Li, Zixuan Liu, Zhenwei Song, Baoyue Zhang, Cheng Peng, Entao Li, Yilong Yang, Jianmin Li, Sandra Chiu, Changming Yu

**Affiliations:** ^1^ School of Medicine Zhejiang University Hangzhou 310058 China; ^2^ Laboratory of Advanced Biotechnology Beijing Institute of Biotechnology Beijing 100071 China; ^3^ State Key Laboratory of Virology Wuhan Institute of Virology Center for Biosafety Mega‐Science Chinese Academy of Sciences Wuhan 430207 China; ^4^ University of Chinese Academy of Sciences Beijing 100049 China; ^5^ Division of Life Sciences and Medicine University of Science and Technology of China Hefei 230027 China

**Keywords:** antibodies, cross‐neutralizing, fusion glycoprotein, henipaviruses, protection, vulnerabilities

## Abstract

Hendra and Nipah viruses (HNVs), zoonotic paramyxoviruses with >50% case fatality rates, cause fatal encephalitis and respiratory disease, yet lack approved therapies. Here, nine rhesus‐derived monoclonal antibodies (mAbs) targeting the fusion glycoprotein (F) prefusion conformation are developed. Four mAbs exhibit first‐rate cross‐neutralization against HNVs, with two showing synergistic potency when combined with attachment glycoprotein (G)‐specific mAbs. Single‐dose administration of mAbs confers robust protection against lethal Nipah virus challenge in hamsters. Structural insights reveal that 8 of the 9 potent mAbs adopt a human IGHV4‐59‐like framework with protruding CDRH3 loops, forming pushpin‐shaped paratopes that stabilize the prefusion F‐trimer by occupying vulnerable interprotomer cavities. Systematic mutational profiling identifies 14 prefusion‐locking residues within the F ectodomain, classified as i) structural linchpins governing fusogenicity or ii) immunodominant hotspots targeted by cross‐neutralizing mAbs. This work delivers promising therapeutic candidates against HNVs and provides blueprints for the rational design of antibodies and vaccines targeting viral fusion machinery.

## Graphical abstract

Hendra and Nipah viruses (HNVs) pose significant threats to global health. This work reports potent cross‐neutralizing antibodies targeting the fusion glycoprotein (F) and reveals shared features of these antibodies and vulnerabilities of F, thereby guiding the rational design of countermeasures against HNVs and similar pathogens.

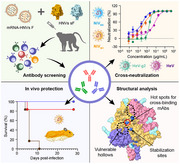

## 1 Introduction

Henipaviruses, zoonotic members of the family *Paramyxoviridae*, are non‐segmented, negative‐sense RNA viruses with significant public health implications. Following the initial identification of the Hendra viruses (HeV) in the 1990s, over a dozen henipavirus‐like viruses have been discovered.^[^ ^1^, ^2^, ^3^, ^4^, ^5^, ^6^, ^7^, ^8^, ^9^ ^]^ In the latest update to the virus taxonomy of paramyxoviridae by the International Committee on Taxonomy of Viruses, several of these, including Mojiang virus (MojV) and Langya virus (LayV), have been classified under the newly established genus *Parahenipavirus*.^[^ ^10^ ^]^ The genus *Henipavirus* currently comprises Hendra virus, Nipah virus (NiV), Cedar virus, Ghana virus, and Angavokely virus. Among the five recognized species, Hendra and Nipah viruses (HNVs) are the most pathogenic, causing severe respiratory and encephalitic symptoms in humans, with case fatality rates ranging from 50% to 100%.^[^ ^11^, ^12^ ^]^ NiV comprises two major clades: the Malaysia strain (NiV_MY_) and the Bangladesh strain (NiV_BD_).^[^ ^13^ ^]^ A new genotype of HeV designated HeV‐g2, which exhibits 84% sequence identity to the original isolate at the nucleotide level, has been recently reported.^[^ ^14^, ^15^ ^]^ Multiple species of the genus *Pteropus* serve as natural reservoir hosts for HNVs.^[^ ^16^ ^]^ HNVs can spill over into many mammalian species, including pigs, cows, goats, horses, dogs, cats, and humans.^[^ ^11^, ^17^, ^18^, ^19^, ^20^, ^21^, ^22^, ^23^ ^]^ Transmission of HNVs to humans is primarily associated with close contact with infected animals or consumption of contaminated foods.^[^ ^13^, ^18^ ^]^ Moreover, the presence of virions in lung and nasopharyngeal secretions during acute human infections, along with evidence of droplet transmission in hamster models, suggests that HNVs have the potential to cause pandemics.^[^ ^24^, ^25^ ^]^ Although the high fatality rate may limit widespread transmission within human populations, the circulation of unknown strains and the emergence of more contagious variants through mutations could further increase the risk of future outbreaks. Based on previous outbreaks and the distribution of *Pteropus* bats, approximately one‐quarter of the global population resides in regions at risk for HNV emergence. Due to the epidemic potential and the lack of effective countermeasures, Nipah and henipaviral diseases are prioritized on the World Health Organization (WHO) list for research and development. However, no vaccines or therapeutics have been approved for human use.

HNV infections occur annually and seasonally in South and Southeast Asia. According to the Disease Outbreak News of the WHO, the last three HNV‐related outbreaks (2023.01–2024.02), all caused by NiV, occurred in Bangladesh, with 11 (8), 6 (2), and 2 (2) cases (deaths) reported, respectively. Advancing vaccine development and stockpiling is essential, and several vaccine candidates, such as HeV‐sG‐V, PHV02, and mRNA‐1215, are currently being evaluated in clinical trials.^[^ ^26^ ^]^ However, in the context of minor, sporadic, and regional outbreaks, therapeutic drugs may be a more appropriate coping strategy against HNVs.

HNV invasion relies on a series of spatiotemporal sequences involving the attachment glycoprotein (G) and fusion glycoprotein (F), including G binding to ephrin‐B2/B3 receptors, G–F interactions, and virus‐host membrane fusion triggered by F conformational changes.^[^ ^27^, ^28^ ^]^ Given their essential roles in entry, G and F are the primary targets for antibody development. The first reported and most studied monoclonal antibody (mAb), m102.4, an affinity‐mature derivative of a clone obtained from the human nonimmune antibody library,^[^ ^29^ ^]^ recognizes the receptor‐binding domain (RBD) of G^[^ ^30^ ^]^ and exhibits robust protective efficacy against HNV infections in ferrets and non‐human primates.^[^ ^31^, ^32^, ^33^ ^]^ Compassionate use of m102.4 has successfully protected 16 individuals at high HNV exposure risk,^[^ ^26^ ^]^ and its safety and tolerability were evaluated in a phase I trial.^[^ ^34^ ^]^ 5B3 is the first reported antibody that recognizes the prefusion conformation of the F protein.^[^ ^35^, ^36^ ^]^ Its humanized derivative, h5B3.1, completely protected NiV‐ or HeV‐infected ferrets when administered at two doses post‐exposure.^[^ ^37^ ^]^ Recently, several potent neutralizing antibodies targeting G (e.g., HENV‐26/32,^[^ ^38^ ^]^ HENV‐103/107,^[^ ^39^ ^]^ n/hAH1.3,^[^ ^40^ ^]^ and 1E5/1B6^[^ ^41^ ^]^) or F (e.g., mAb66,^[^ ^42^ ^]^ 12B2/1F5,^[^ ^43^, ^44^ ^]^ and 4H3/1H8^[^ ^45^ ^]^) have been reported, further expanding the pool of drug candidates for HNV infections.

Compared to the asymmetrical and flexible G‐tetramer,^[^ ^46^ ^]^ the F‐trimer shares a more conserved sequence and compact structure among species of the genus,^[^ ^47^, ^48^, ^49^ ^]^ facilitating antibody recognition of each monomer and increasing the likelihood of cross‐reactivity. However, several limitations exist in current studies on F and its antibodies: 1) Existing F‐specific antibodies, including 5B3, are murine‐derived,^[^ ^43^, ^45^ ^]^ potentially causing immunogenicity in therapeutic applications; 2) No direct comparisons of neutralization or protection efficacy have been made between F and G antibodies, and some antibodies also lacked cross‐reactivity evaluation against HNVs;^[^ ^45^ ^]^ 3) Although diverse F protein epitopes have been identified, the most vulnerable epitopes relevant to protection remain undefined due to the lack of in vivo data;^[^ ^43^, ^45^ ^]^ 4) The critical functional and structural sites of F at the full‐length scale remain unclear.

Herein, we isolated a panel of F‐specific antibodies using a sequential immunization strategy with mRNA vaccines and recombinant antigens. These antibodies recognized the natural structure of F, demonstrated cross‐reactivity and high avidity, and exhibited potent neutralizing activity against both pseudotyped and authentic HNVs. Several antibodies were comparable to or surpassing the most potent G‐specific antibodies in vitro. We determined the cryo‐electron microscopy (cryo‐EM) structures of the NiV F in complex with the two most potent cross‐neutralizing antibodies (cnAbs), 1D6 and 5C8, revealing the pushpin‐shaped paratope structures and hollow‐filling binding modes. The three representative antibodies significantly protected the hamsters challenged with lethal NiV. Moreover, we identified the critical residues for maintaining the fusion function and prefusion conformation through alanine scanning mutations spanning the entire F ectodomain. These antibodies represent promising therapeutic candidates. The characterization of vulnerable epitopes and critical functional sites of F provides essential information for the rational design of antibodies and vaccines against HNVs.

## 2 Results

### 2.1 HNV CnAbs were Isolated from an Immunized Rhesus Monkey

**Figure 1:**
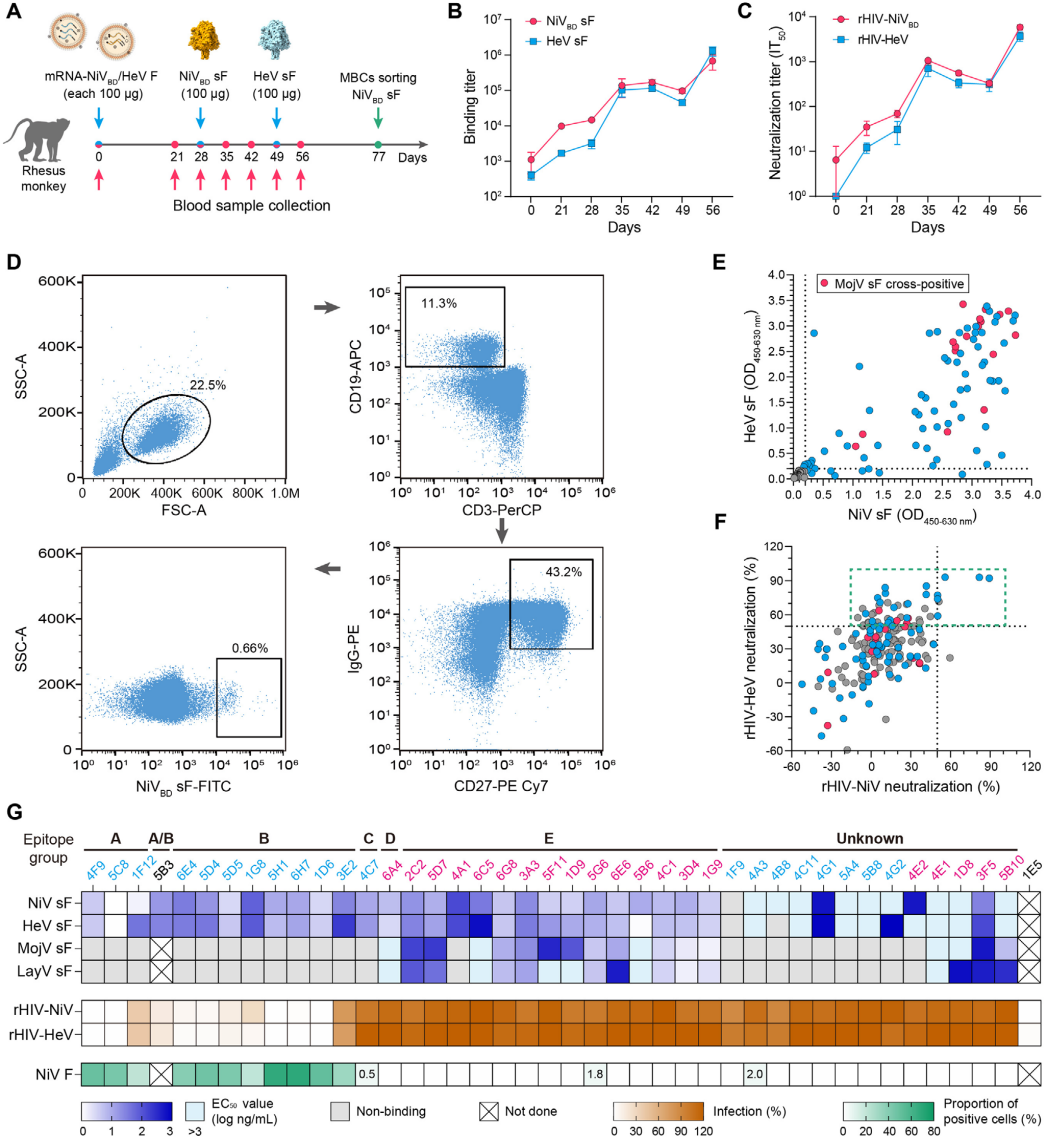
HNVs cross‐neutralizing antibodies isolated from an immunized rhesus monkey. A) Schematic of the experimental workflow for immunizations (blue arrows), serum collection (magenta arrows), and memory B cells sorting (green arrow). B) The serum binding titer to the NiV_BD_ or HeV sF is tested by ELISA (n = 3). Data are presented as mean ± SD. C) The half maximal inhibitory titer of the serum against rHIV‐NiV_BD_ or ‐HeV (n = 3). The value is defined as 1 when half inhibition cannot be achieved using undiluted serum. Data are presented as mean ± SD. D) Single NiV_BD_ sF‐specific memory B‐cell sorting. Gating strategies and cell proportions are indicated. E,F) Identification of HNVs binding/neutralizing antibodies using the supernatants of linear expression cassettes. Dots in gray, bright blue, and magenta indicate non‐binding, NiV or HeV sF positive (>0.2), and MojV sF cross‐reactive clones, respectively. Clones achieving >50% neutralization (framed by the dashed green boxes) and those demonstrating cross‐reactivity with MojV sF are selected for purification. G) Characterization of 40 antibodies (n = 3): binding to recombinant sF, neutralization against rHIV‐HNVs, binding to membrane‐anchored full‐length of NiV F, and binding competitiveness. HNVs cross‐neutralizing antibodies targeting F (5B3) and G (1E5) were used as controls.

Recent studies on COVID‐19 vaccines have shown that the “mix‐and‐match” strategy can elicit more desirable protection and humoral immunity.^[^ ^50^, ^51^, ^52^, ^53^ ^]^ To obtain potent cnAbs, we sequentially inoculated a rhesus monkey with mRNA‐NiV_BD_/HeV F‐LNP (mRNA vaccines encoding the full‐length NiV_BD_ or HeV F protein), NiV_BD_ sF (recombinant prefusion stabilized F ectodomain trimer), and HeV sF on days 0, 28, and 49, respectively (**Figure** **1**A). We collected serum from the animal at seven‐time points and titrated the levels of humoral immunity. We observed similar growth trends in serum binding antibodies for NiV_BD_ sF and HeV sF, as well as in IC_50_ (half maximal inhibitory concentration) titers against rHIV‐NiV_BD_ (human immunodeficiency virus skeleton pseudovirus carrying NiV_BD_ G and F glycoproteins) and rHIV‐HeV (Figure 1B,C). These findings are consistent with the high sequence and structural conservation between the two viral F proteins.^[^ ^48^ ^]^ Following the second booster immunization, the binding and neutralizing antibodies against HNVs increased by ≈1000‐fold.

We isolated peripheral blood mononuclear cells (PBMCs) from blood samples on day 77 and sorted a total of 564 antigen‐specific memory B cells using a fluorescent cocktail of CD3^−^CD19^+^CD27^+^IgG^+^NiV_BD_ sF^+^ (Figure 1D). We cloned the recovered 131 VH‐Vκ and 61 VH‐Vλ pairs into linear expression cassettes to construct full‐length heavy‐ or light‐chain genes of chimeric mAbs, incorporating the constant region of human IgG1. Screening of transient transfection supernatants identified 90 clones positive for NiV_BD_ or HeV sF, with unique genes encoding diverse V/J sequences and combinations (Figure S1, Supporting Information). Most clones (88/90) reacted with both HNV sFs, and 20 of which also cross‐bound to recombinant MojV sF (Figure 1E). Based on their neutralization (capable of neutralizing rHIV‐NiV_BD_/HeV by more than 50%) and cross‐binding capacity (cross‐reactive with MojV sF), we selected 40 clones for further purification and analysis (Figure 1E,F). Most (39/40) purified mAbs retained binding ability against HNV sF, although eight mAbs exhibited relatively weak binding for both HNV sFs (Figure 1G). All mAbs cross‐reactive with MojV sF also reacted with LayV sF. In quantitative neutralization assays conducted at a 0.5 µg mL^−1^ concentration, 11 mAbs demonstrated cross‐neutralization activity against rHIV‐HNVs (Figure 1G). Among these, nine and five antibodies exhibited neutralization potency comparable to or more potent than that of cnAbs 5B3^[^ ^36^ ^]^ (anti‐F) and 1E5^[^ ^41^ ^]^ (anti‐G), respectively.

To delineate the epitope specificity of these mAbs, we performed a competitive enzyme‐linked immunosorbent assay (ELISA). The relative binding percentage of a biotinylated secondary antibody to NiV_BD_ sF was measured in the presence of each primary antibody or an irrelevant antibody (at 100‐fold the concentration of the biotinylated antibody). Based on the competitive binding values, 11 cross‐neutralizing mAbs primarily clustered into two distinct groups (Figure 1G; Figure S2, Supporting Information). Three mAbs, 4F9, 5C8, and 1F12, competed with 5B3, which targets the junction of domains I and III in the middle of the F‐trimer.^[^ ^36^ ^]^ The epitopes of eight mAbs, 6E4, 5D4, 5D5, 1G8, 5H1, 6H7, 1D6, and 3E2, overlapped with 4H3, which binds to the apex of the F‐trimer.^[^ ^45^ ^]^ To elucidate factors influencing antibody neutralization activity, we further evaluated the binding capacity of these antibodies to NiV F displayed on the membrane surface. Our results indicated a significant positive correlation (r = 0.9450, *P* < 0.0001) between the neutralization potency of antibodies against rHIV‐NiV and their ability to recognize the natural conformation of F (Figure S3A, Supporting Information), and only 11 neutralizing antibodies effectively bound to membrane‐anchored F (Figure 1G). Given their clustering into a single epitope group, inability to neutralize rHIV‐HNVs, lack of binding to native NiV F, and low sequence homology (amino acid identity < 20%) of the F protein between genera, we hypothesize that antibodies cross‐reactive with MojV or LayV sF may recognize artificially integrated C‐terminal structures. This hypothesis was confirmed by negative‐stain electron microscopy analysis of the complex of 1G9 Fab‐NiV sF (Figure S3B, Supporting Information).

### 2.2 CnAbs Exhibited Superior Neutralization Potency against HNVs

**Figure 2:**
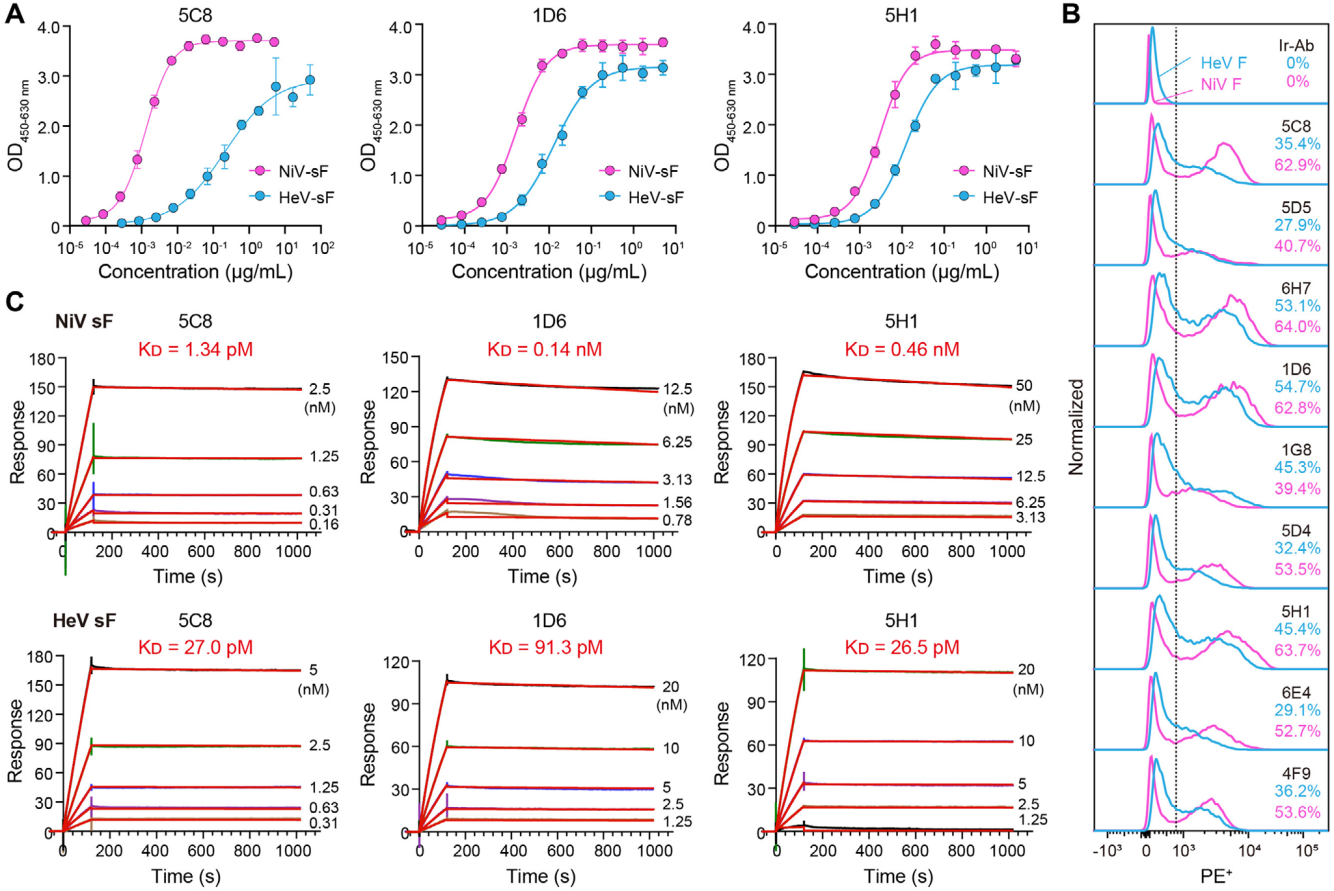
Cross‐binding mAbs recognize two major epitope groups of the natural structure of F. A) Binding curves of cross‐reactive antibodies to NiV or HeV sF determined using ELISA (n = 3). Data are presented as mean ± SD. B) Recognition of antibodies to the full length of NiV_BD_ or HeV F anchored on the 293T cell surface. An irrelevant mAb is used as the negative control, and all curves are normalized (n = 3). Data are presented as mean ± SD. C) Binding kinetics of mAbs to HNVs sF determined using SPR. Five representative curves of each mAb are fitted to compute the kinetic constants.

Given the relatively poor neutralization capacity of 1F12 and 3E2 (Figure 1G), we proceeded to characterize the remaining cnAbs. Nine potent cnAbs featured a long heavy‐chain complementarity‐determining region 3 (CDRH3) length (18–23 aa), significantly exceeding the average length (16.4 aa) of all positive clones (Figure S3C,D, Supporting Information). These cnAbs efficiently bound to HNV sF, with the half‐maximal effective concentrations (EC_50_) ranging from 1.3 to 44.7 ng mL^−1^ for NiV_BD_ sF and slightly higher values from 12.4 to 380.7 ng mL^−1^ for HeV sF (**Figure** **2**A, **Table** **1**; Figure S3E, Supporting Information). Notably, four cnAbs, 6H7, 1D6, 5H1, and 4F9, demonstrated stronger cross‐reactivity than previously reported F‐specific antibodies, 4H3, 1H8, and h5B3.1.^[^ ^36^, ^45^ ^]^ All cnAbs recognized membrane‐displayed HNV F‐trimers, with the proportion of positive cells ranging from 39.4% to 64.0% for NiV F and 27.9% to 54.7% for HeV F (Figure 2B). Among them, three apex binders, 6H7, 5H1, and 1D6, demonstrated the highest binding efficiency to the natural HNV F structure. Surface plasmon resonance (SPR) was employed to evaluate the binding kinetics of cnAbs to NiV_BD_ and HeV sF. All mAbs exhibited nanomolar to picomolar avidity to HNVs sF, with K_D_ values ranging from 1.26 pM to 0.8 nM for NiV sF and 26.5 pM to 1.87 nM for HeV sF (Figure 2C, Table 1; Figure S4, Table S1, Supporting Information). Consistent with ELISA data, most mAbs exhibited weaker binding to HeV sF than NiV sF.

**Table 1:** Summary of binding and neutralizing activity of antibodies.

Clone	Binding EC_50_ ^a)^ [ng mL^−1^]		Natural F [%]		Kinetic constant [M]		Pseudovirus neutralization IC_50_ ^b)^, [ng mL^−1^]				Live virus neutralization IC_50_, [µg mL^−1^]		
	NiV‐sF	HeV‐sF	NiV	HeV	NiV‐sF	HeV‐sF	NiV_BD_	NiV_MY_	HeV	HeV‐g2	NiV_BD_	NiV_MY_	HeV
5C8	1.3	211.4	62.9	35.4	1.34E‐12	2.70E‐11	28.6	130.2	43.1	4.2	0.45	0.02	0.01
4F9	1.7	17.8	53.6	36.2	1.26E‐12	6.45E‐11	54.9	95.0	21.7	1.2	1.04	0.11	0.07
5D4	10.4	380.7	53.5	32.4	8.07E‐10	1.87E‐09	63.7	29.5	30.6	14.1	12.64	2.26	0.97
5D5	8.2	300.3	40.7	27.9	7.19E‐10	1.32E‐09	57.4	96.4	15.8	11.2	12.04	3.32	0.26
6H7	2.6	15.0	64.0	53.1	2.60E‐12	1.64E‐10	19.4	70.6	7.4	1.7	1.60	0.12	0.05
1D6	1.6	12.7	62.8	54.7	1.36E‐10	9.13E‐11	17.8	53.8	3.5	2.0	0.08	0.04	0.05
1G8	44.7	278.7	39.4	45.3	1.59E‐10	7.87E‐10	421.9	520.0	97.2	57.4	0.26	0.09	0.11
5H1	3.0	12.4	63.7	45.4	4.60E‐10	2.65E‐11	6.7	25.5	1.0	1.5	0.48	0.18	0.11
6E4	10.9	367.4	52.7	29.1	4.16E‐10	1.19E‐09	36.1	88.4	12.5	16.7	6.88	0.35	0.25
1H8	4.5	29.2	ND^d)^	ND	ND	ND	8.4	33.0	24.0	103.1	6.68	0.54	0.25
4H3	5.7	/^c)^	ND	ND	ND	ND	11.7	47.7	/	/	22.54	0.20	/
5B3	6.7	98.2	ND	ND	ND	ND	229.8	807.7	143.6	20.5	15.94	1.08	0.3

^a)^ EC_50_, the half‐maximal effective concentration in ELISA;

^b)^ IC_50_, the half‐maximal inhibitory concentration in the neutralization tests;

^c)^ /, non‐binding or non‐neutralizing;

^d)^ ND, not done.

**Figure 3:**
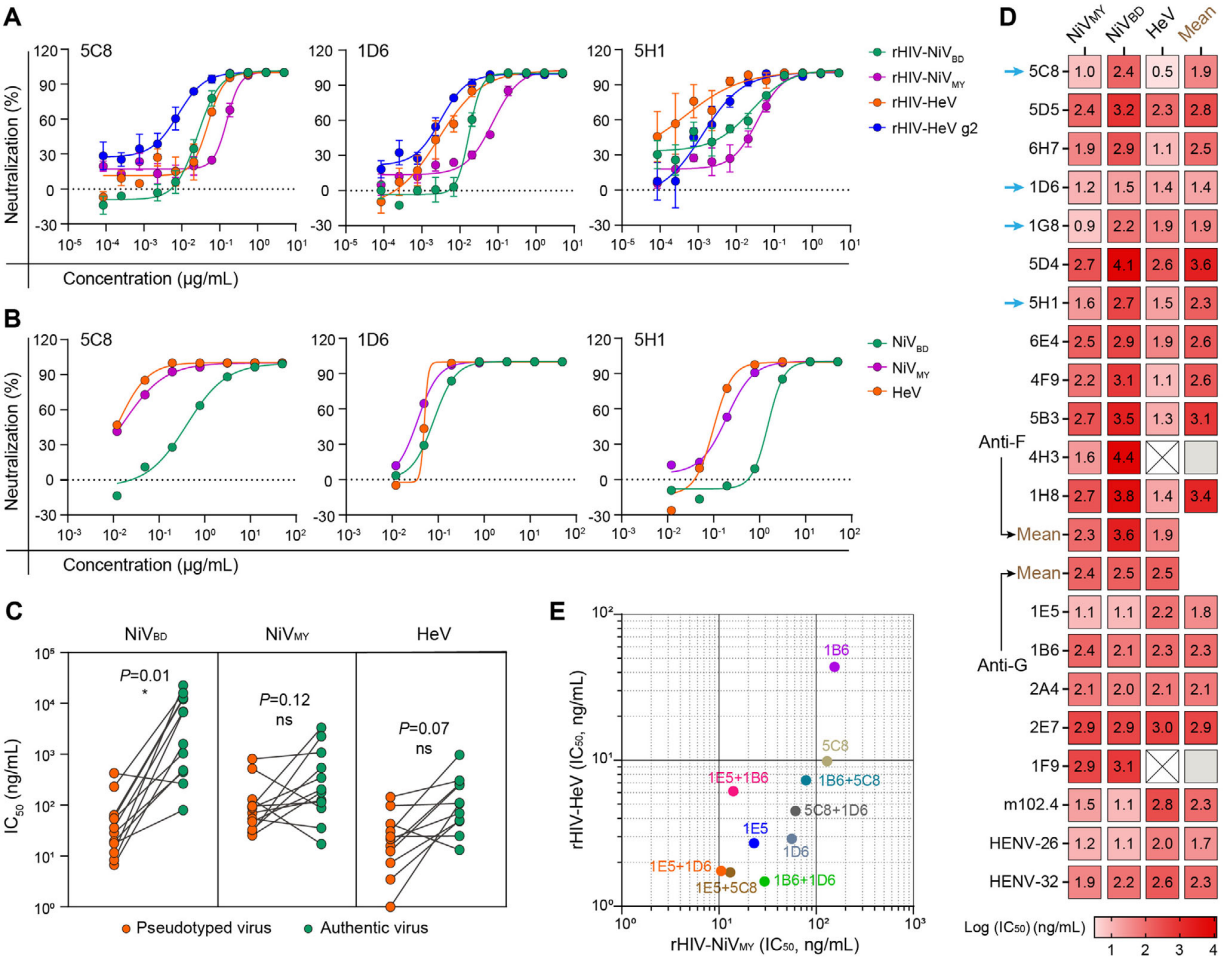
CnAbs exhibit potent neutralizing capacity and significant protection efficacy against HNVs. A) Neutralization curves of mAbs against pseudotyped rHIV‐NiV_BD_, ‐NiV_MY_, ‐HeV, or ‐HeV‐g2 (n = 3). Data are presented as mean ± SD. B) Neutralization curves of mAbs against authentic NiV_BD_, NiV_MY_, or HeV (n = 2). C) Difference analysis of neutralizing activities of antibodies against the pseudotyped and live HNVs (n = 12 for NiV and n = 11 for HeV). The neutralizing IC_50_ values of each antibody against pseudotyped or authentic viruses are indicated as orange and green dots, respectively. The paired t‐test is used to compute the two‐tailed *P* value of the difference in the neutralization ability of antibodies against pseudotyped and live viruses of the same species. *P < 0.05. D) The half maximal inhibitory concentrations (IC_50_) of mAbs against HNVs. The red‐to‐white gradient squares indicate logarithm values of IC_50_ (ng/mL), and white squares with diagonal lines indicate that the IC_50_ was not reached at 50 µg mL^−1^ concentration. The light gray squares represent that the average IC_50_ value cannot be computed owing to the non‐cross‐neutralization, and blue arrows indicate the four F‐specific antibodies with the lowest average IC_50_ value. E) IC_50_ values of mAbs or mAb combinations against pseudotyped rHIV‐NiV_MY_ or ‐HeV (n = 3). Data are presented as mean of three replicates.

To assess the neutralization potency and breadth of the antibodies, we performed dose‐response inhibition assays using four replication‐deficient rHIV‐pseudotyped HNVs (**Figure** **3**A; Figure S5A, Supporting Information). As anticipated, all the cnAbs demonstrated potent anti‐HNV activity. Specifically, eight antibodies, excluding 1G8, effectively cross‐neutralized rHIV‐NiV_BD_/NiV_MY_ and rHIV‐HeV/HeV‐g2 with IC_50_ values below 0.15 µg mL^−1^ (1 nM). The average neutralization capacity of five apex binders (6H7, 1D6, 5H1, 5D4, and 6E4) was more potent than the middle binders. Notably, most cnAbs exhibited more vigorous neutralization against rHIV‐HeVs than rHIV‐NiVs (Figure 3A, Table 1; Figure S5A, Supporting Information), albeit with relatively weaker binding activity and avidity toward HeV sF (Table 1). Such phenomena may stem from inherent disparities in the stability of native F or be attributed to the discrepancy in the structural fidelity of recombinant sF.

We evaluated the neutralization efficacy of the cnAbs against authentic HNVs using plaque‐reduction neutralization tests. All cnAbs maintained their neutralizing breadth against the HNVs (Figure 3B; Figure S5B, Supporting Information). Unexpectedly, we observed chaotic pseudo‐authentic virus neutralization mapping relationships (Figure 3C). For NiV_BD_, all cnAbs, except 1G8, exhibited a significant reduction in neutralization activity. The IC_50_ values of three cnAbs (5D4, 5D5, and 6E4) and two control cnAbs (1H8 and 4H3) increased by ≈200 to 2000‐fold. In contrast, the antibodies largely preserved their neutralizing capacity toward the other two viruses, with IC_50_ variations ranging from 0.2 to 34.4‐fold, except for 5D4 (76.6‐fold increase against NiV_MY_) and 5H1 (110.0‐fold increase against HeV). Remarkably, several cnAbs demonstrated enhanced neutralization potency against authentic viruses, including 1G8 against NiV_BD_, 1G8, 5C8, and 1D6 against NiV_MY_, and 5C8 against HeV. Four cnAbs, 5C8, 1D6, 1G8, and 5H1, exhibited exceptionally potent neutralizing activity against the three HNVs, with IC_50_ values below 0.75 µg mL^−1^ (5 nM) (Table 1).

Next, we compared the neutralization potency of the 12 mAbs detailed above with that of eight previously reported representative G‐specific potent neutralizers. Overall, the neutralizing efficacy of G‐targeting mAbs against NiV_MY_, NiV_BD_, and HeV was comparable (0.27 vs 0.21 µg mL^−1^), superior (0.30 vs 4.22 µg mL^−1^), and inferior (0.35 vs 0.08 µg mL^−1^) to that of F‐specific mAbs, respectively (Figure 3D; Figure S5C, Supporting Information). The cnAb 5H1 (0.21 µg mL^−1^) exhibited equivalent mean neutralization capacity to m102.4. Two cnAbs, 1G8 (0.08 µg mL^−1^) and 5C8 (0.09 µg mL^−1^), were slightly less potent than 1E5 (0.06 µg mL^−1^) and HENV‐26 (0.05 µg mL^−1^). Among the tested antibodies, cnAb 1D6 had the lowest average IC_50_ values (0.02 µg mL^−1^) and effectively neutralized the three viruses at a comparably low concentration.

Combinatorial neutralization efficacy was systematically evaluated using four non‐overlapping cnAbs targeting distinct epitopes: G‐specific (1E5, 1B6) and F‐specific (1D6, 5C8). In the fixed‐ratio methodology, combinations of subpotent cnAbs (1B6 or 5C8) with other antibodies, whether targeting the same or different antigens, enhanced their neutralizing potency. However, only heterologous combinations further augmented the neutralizing activity of the potent parent cnAbs (1E5 or 1D6). Among the combinations, G‐RBD‐targeting 1E5 paired with F‐targeting 1D6 or 5C8 exhibited the most potent cross‐neutralizing activity (Figure 3E; Figure S6A, Supporting Information). Due to the neutralization saturation of these antibodies against pseudoviruses, synergism was only evident at low concentrations in the dose‐response matrix methodology for 1B6 and 1D6, 1E5 and 1D6, and 1E5 and 5C8 (Figure S6B, Supporting Information). The synergistic potential of these cnAbs warrants further investigation using authentic viruses in more rigorous assays.

### 2.3 Potent CnAbs Significantly Protected Hamsters from NiV_MY_ Infections

**Figure 4:**
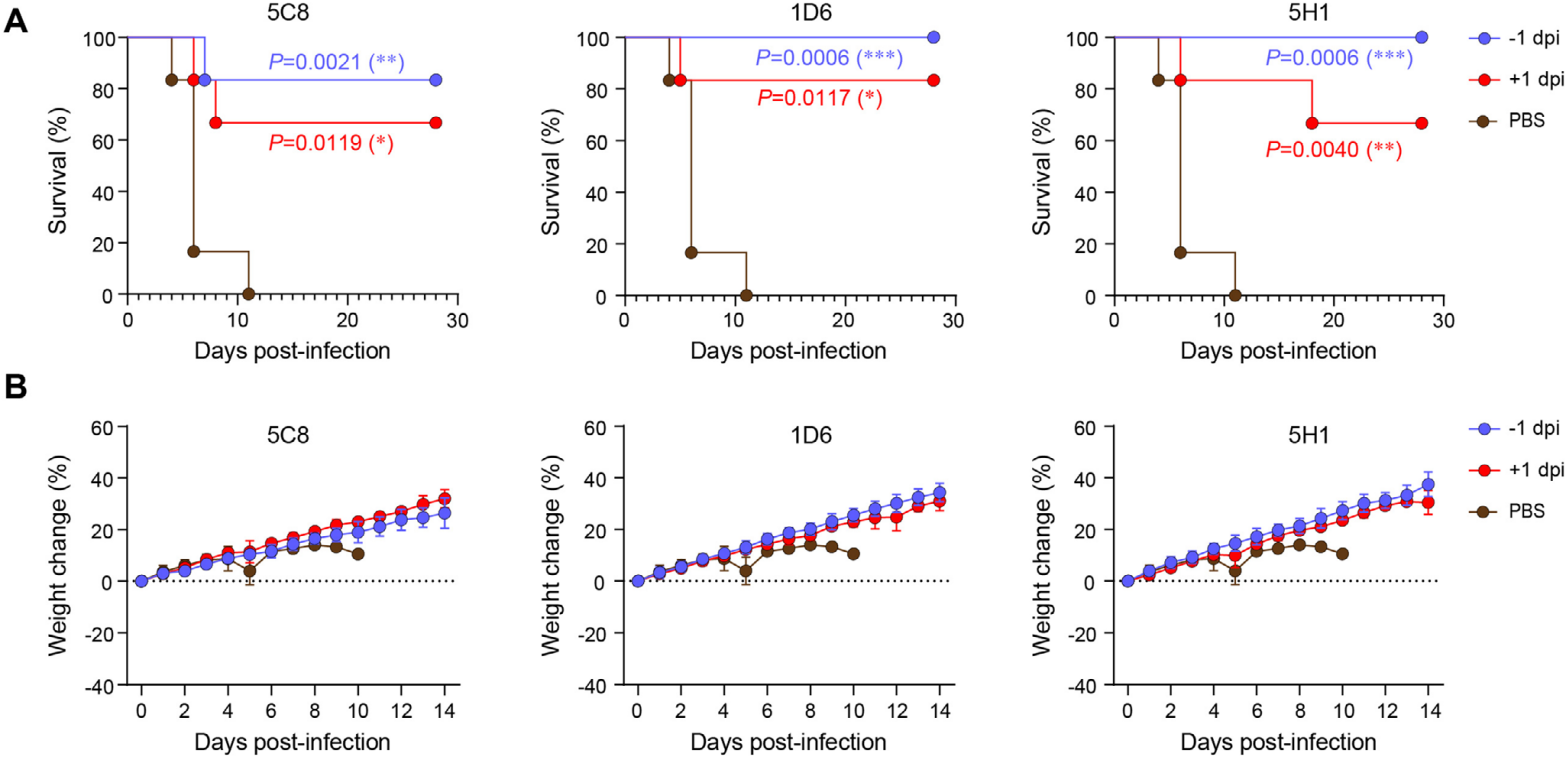
Potent cnAbs significantly protect hamsters from NiV_MY_ infections. A) Kaplan–Meier survival curve of hamsters infected with NiV_MY_. Hamsters (n = 6) are prophylactically or therapeutically treated with 1 mg antibody via the intraperitoneal route, and the *P* values are determined using the Mantel–Cox log‐rank test. *P < 0.05, **P < 0.01, and ***P < 0.001. B) Weight change of hamsters infected with NiV_MY_.

Given the suboptimal productivity of 1G8, we selected three candidate cnAbs with the most potent cross‐neutralizing activity (Figure 3D)—namely 5C8, 1D6, and 5H1—to evaluate their in vivo efficacy against HNV infections. Disease severity in HNV‐infected Syrian golden hamsters (SGH; *Mesocricetus auratus*) is dependent on the virus/strain, route of infection, and dose. Specifically, the challenge with NiV_MY_ via the intraperitoneal (i.p.) route has resulted in uniform lethality.^[^ ^54^, ^55^ ^]^ This study utilized a highly stringent SHG model, inoculating animals with 1000 LD_50_ of NiV_MY_ via the i.p. route. We administered a single 10 mg kg^−1^ dose of each candidate cnAb intraperitoneally, either 24 h before or after infection, and monitored weight change for 14 days and survival for 28 days. In the control group, five of the six animals died on day 6, and the other died on day 11 (**Figure** **4**A), consistent with the two‐stage pattern of disease progression in hamsters.^[^ ^39^ ^]^ All cnAb interventions provided significant protection, with normal weight gain observed throughout the monitoring period (Figure 4B). For prophylactic administration, 1D6 and 5H1 conferred complete protection, whereas one animal in the 5C8 group succumbed to infection. For therapeutic administration, the survival rate was 83.3% (5/6) in the 1D6 group. Although the survival rate was the same (66.7%) in the other two treatment groups, 5H1 extended the average survival time of deceased hamsters to 12 days, compared with 7 days for 5C8 (Figure 4A). Collectively, these three potent cnAbs, particularly the two apex binders, demonstrated robust in vivo efficacy and hold promise as therapeutic agents for HNV infections.

### 2.4 Long CDRH3 of the Two Most Potent CnAbs Filled the Hollows Formed by the Adjacent Protomers of F‐trimer

To elucidate the structural basis of neutralization, we selected the most potent cnAbs, 1D6 and 5C8, from the two epitope groups and determined the structures of antibody‐antigen (Ab‐Ag) complexes using cryo‐EM. The structures of the 1D6 and 5C8 Fab complexes with NiV_BD_ sF‐trimer were solved at resolutions of 1.99 and 2.22 Å, respectively (Figures S7 and S8, Table S2, Supporting Information).

**Figure 5:**
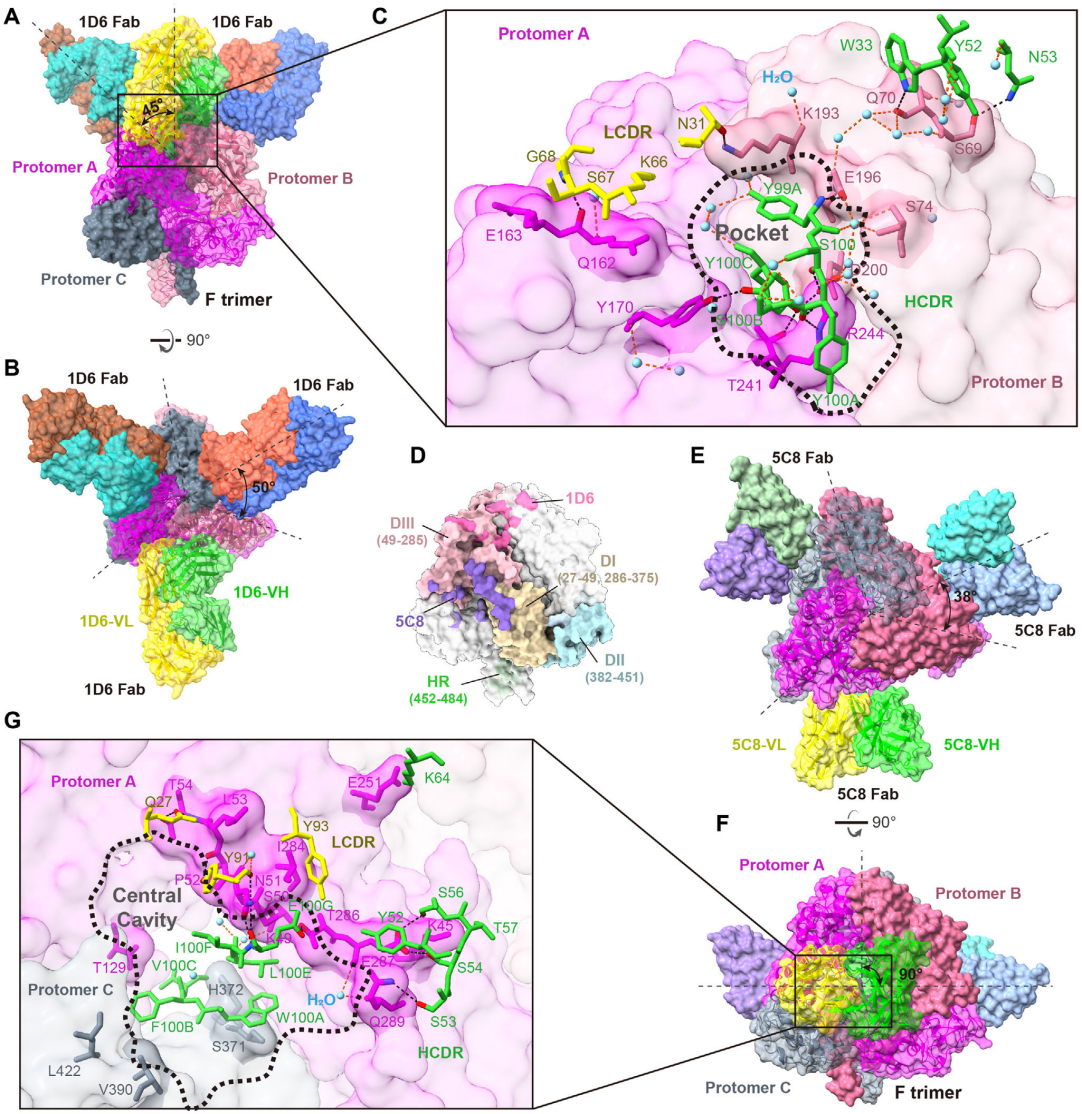
Long CDRH3 of the two most potent cnAbs fill the hollows formed by the adjacent protomers of F. A,B) Front (A) and top (B) views of the cryo‐EM structure of 1D6 Fab in complex with NiV_BD_ sF. Protomers of F‐trimer are colored in magenta, pale violet red, and slate gray, respectively. A representative Fab and the two protomers of F to which it binds are depicted as ribbon diagrams beneath a transparent surface. C) Magnified view of the interface between 1D6 and F‐trimer. The NiV sF is represented as a molecular surface, and the epitopes of 1D6 are highlighted. Interface residues are shown as stick representations. The CDRH, CDRL, water molecule, oxygen atom, and nitrogen atom are colored in green, yellow, sky blue, red, and blue, respectively. Direct Ab‐Ag H‐bonds and water‐mediated H‐bond interactions are represented by black and orange dashed lines, respectively. D) Structure diagram of the prefusion NiV F. The domain I‐III (DI‐III), heptad repeat (HR), 1D6 epitope, and 5C8 epitope on a protomer are colored wheat, light blue, light pink, lime green, hot pink, and medium purple, respectively. E–G) Top (E), front (F), and interface zoomed view (G) of the 5C8 Fab in complex with NiV_BD_ sF.

Three 1D6 Fabs bound to the F‐trimer apex at a 45° angle, with each Fab rotated by ≈50° relative to the F trimer's 3‐fold symmetry axis (**Figure** **5**A,B). A single 1D6 Fab covered an area of 850.3 Å^2^ on the F surface via interactions involving CDRH1–3 and CDRL1–2, with two adjacent protomers nearly equally sharing the antibody footprint (Figure 5C,D). At the contact interface with a threshold of 3.5 Å, a limited number of residues—12 from the antibody and 11 from the antigen—formed a robust interaction network comprising up to 11 hydrogen bonds (H‐bonds). Although five of the six CDRs participated in the interaction with F, CDRH3 was likely critical for binding (Figure 5C; Figure S9A, Supporting Information). The tyrosine‐rich “YSYSY” motif within the central CDRH3 loop demonstrated high shape complementarity with a pocket formed by two neighboring F protomers. All five CDRH3 paratope residues were positioned within this binding pocket, engaging in extensive interactions with surrounding residues and contributing 7 of the 11 total H‐bonds identified in the interface. Each residue of the “YSYSY” motif participated in hydrogen bonding, with two tyrosine residues forming more than one H‐bond: the mainchain O atom of Y100A^CDRH3^ interacted with the Nε (donor‐acceptor distance = 3.0 Å) and Nη2 (3.1 Å) atoms of R244^F^ via two H‐bonds; the Oη atom of the deepest immersed Y100C^CDRH3^ formed two H‐bonds with the Oγ1 atom of T241^F^ (3.1 Å) and Oδ1 atom of D200^F^ (2.4 Å) (Figure 5C). Twenty‐three interfacial water molecules were identified at the Ab‐Ag interface. These interfacial waters strengthened the binding by mediating polar interactions via hydrogen‐bonded water bridges or occupying hydrophobic cavities to stabilize the interfacial architecture. Furthermore, the binding pocket exhibited a rich array of π‐driven interactions, comprising six π‐alkyl and one π‐cation bonds, collectively augmenting the overall binding ability.

In the Ab‐Ag complex, 5C8 bound to a quaternary epitope that spans the DI and DIII regions of one protomer and the DII region of another, mediated by CDRH1‐3 and CDRL3 (Figure 5D–G; Figure S9B, Supporting Information). The three Fabs attached to the waist of the F‐trimer at a nearly horizontal angle, with each Fab rotated by ≈38° relative to the trimeric axis of F (Figure 5E,F). The epitope of 5C8 encompassed 17 residues, with 4, 4, and 9 residues originating from the DI, DII, and DIII regions, respectively, burying an area of ≈976.1 Å^2^ (Figure 5G). 5C8 employed a hollow‐filling recognition mechanism analogous to 1D6, characterized by dominant CDRH3 insertion into the F‐trimer central cavity, albeit penetration depth comparatively shallower than 1D6 CDRH3 (Figure S9C,D, Supporting Information). The Ab‐Ag interface comprised 10 H‐bonds, with three key antigen residues contributing seven of these interactions. Specifically: 1) The Nδ2 atom of N51^F^ formed H‐bonds with the mainchain O atoms of Y91^CDRH3^ (3.4 Å) and E100G^CDRH3^ (3.0 Å), while its Oδ1 atom interacted with the N atom of E100G^CDRH3^ (3.1 Å); 2) The N and Oγ1 atoms of T54^F^ made H‐bonds with the Oε1 atom of Q27^CDRL1^ at distances of 3.2 Å and 3.0 Å, respectively; 3) The Oε1 and Oε2 atoms of E287^F^ formed H‐bonds with the Oγ atom of S56^CDRH2^ (3.1 Å) and the Oγ atom of S54^CDRH2^ (3.3 Å), respectively. Unlike 1D6, hydrophobic interactions played an important role in mediating the strong antigen binding of 5C8. The CDRH3 of 5C8 harbored a cluster of hydrophobic residues (Figure S3D, Supporting Information). Five (W100A, F100B, V100C, L100E, and I100F) out of six paratope residues from CDRH3 were hydrophobic and penetrated the central cavity, establishing extensive hydrophobic effects with the complementary pocket formed by F protomers (Figure 5G). Additionally, two of the five structurally solved water molecules at the Ab‐Ag interface mediated water‐bridging interactions: i) between the N atom of L100E^CDRH3^ and the O atom of K49^F^ (3.0‐3.1 Å), and ii) linking the O atom of Y91^CDRL3^ to the O atom of N51^F^ (2.9‐3.1 Å), collectively contributing to complex stabilization.

### 2.5 Hollow‐Filling Recognition Mode Enables CnAbs to Lock F in Prefusion State and Confers High Tolerance to F Mutations

**Figure 6:**
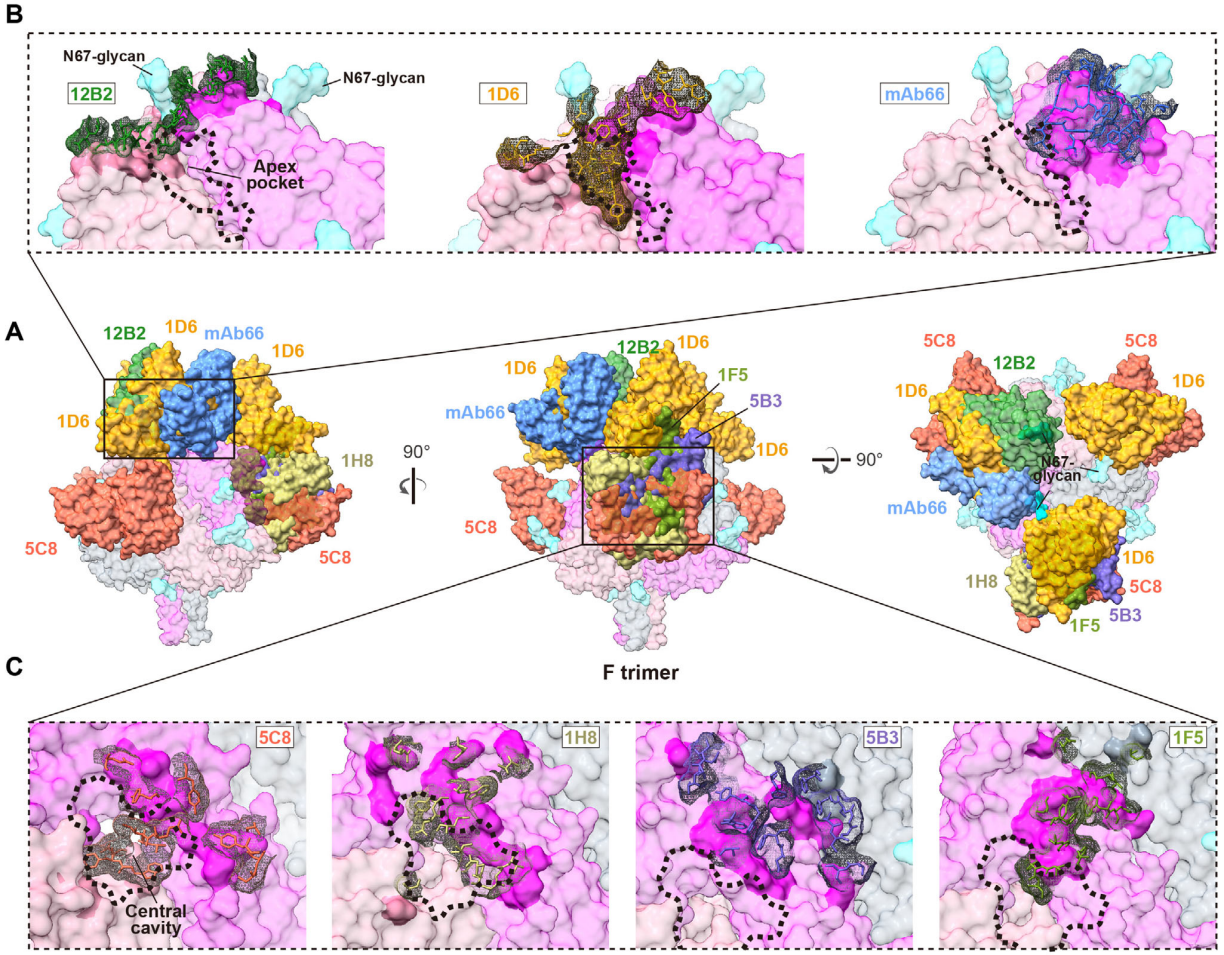
Distinct hollow‐centric engagement of 1D6 and 5C8 compared to reported antibodies that target F‐trimer. A) Superimposition of the 1D6, 5C8, and reported cross‐neutralizing antibodies onto NiV sF. To clarify the spatial position relationships, three Fabs of 1D6 and 5C8 and one Fab of the remaining antibodies are shown. Molecules are shown as surface representations. Protomers of F‐trimer are colored in magenta, pale violet red, and slate gray, respectively. Glycans are colored in cyan, and the colors of the diverse Fabs are consistent with their labels. B,C) Footprints of the apex (B) or middle (C) binders on the F‐trimer. Three F‐trimer protomers are colored in magenta, pale violet red, and slate gray, respectively, and the epitopes of antibodies are highlighted. The paratope residues of antibodies are shown as stick diagrams beneath a mesh surface. Thick black dashed lines indicate the apex pocket and central cavity formed by adjacent protomers.

To determine if the recognition patterns of the two cnAbs profiled were special, we superimposed the structures of known HNVs F‐specific antibodies onto the NiV F‐trimer. Since most (4/5) non‐neutralizing and breadth‐undetermined antibodies exhibited single‐protomer recognition (Figure S9E, Supporting Information), we focused on the binding patterns of cnAbs. 1D6, 12B2 and mAb66 bound to the apex of F, where 5C8, 5B3, 1F5, and 1H8 were classified as middle binders (**Figure** **6**A). All HNVs cnAbs, except mAb66, recognized the multivariate epitopes formed by two neighboring protomers. However, 5B3, 1H8, and 1F5 manifested apparent single protomer dominance (Figure 6B,C). In protomer‐balanced antibodies, the 12B2 epitope crossed with Asn67‐linked oligosaccharides, potentially impairing its actual activity against HNVs (Figure 6A,B). The 1D6 Fab was sandwiched between mAb66 and 12B2 at a lower position relative to the apex, making its binding less susceptible to the Asn67‐linked glycan. Although the epitopes of the three apex binders were located near the apex pocket, only 1D6 was deeply inserted (Figure 6B). The four middle binders appeared to possess more glycan‐free epitopes. Compared with 5C8 and 1H8, 5B3 and 1F5 were higher in the binding position, which generated steric hindrance with 1D6 (Figure 6A), explaining the previously observed competition of 1D6 for 5B3 but not for 5C8 (Figure S2, Supporting Information). The relatively low position made 5C8 and 1H8 closer to the F‐trimer central cavity. Although 1F5 and 1H8 contacted the central cavity, it was predominantly a shallow edge effect and not as deep an immersion or strong interaction as 5C8 (Figure 6C).

**Figure 7:**
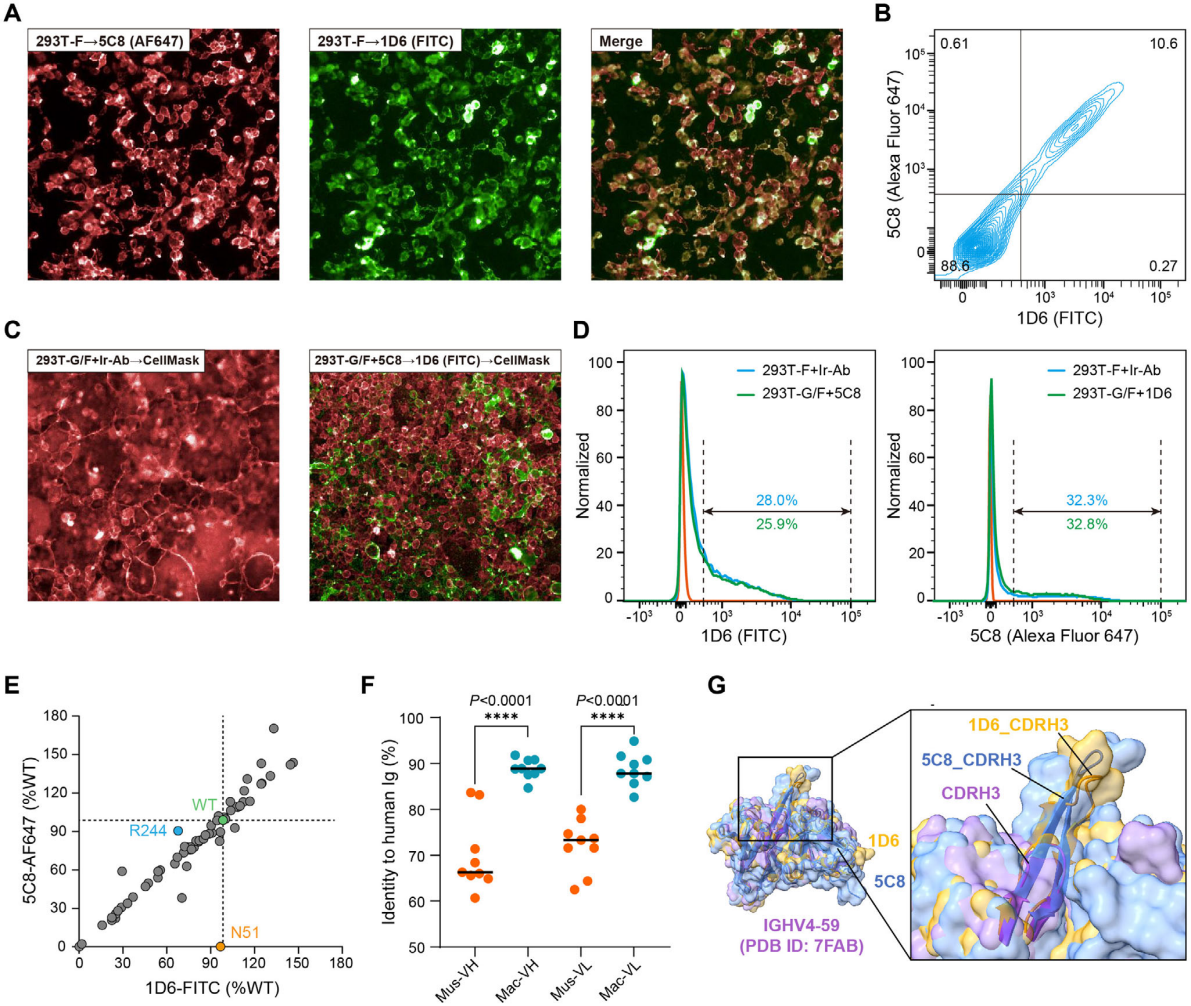
CnAbs with pushpin‐shaped structures lock F in the pre‐fusion state and exhibit high tolerance to F mutations. A,B) Competitive binding of 1D6‐FITC and 5C8‐AF647 to membrane‐displayed F‐trimers assessed by fluorescence imaging (A) and flow cytometry (B). C,D) The cell‐cell fusion (C) and the binding of another non‐competing fluorescence‐labeled antibody (C,D) in cells co‐transfected with G/F in the presence of antibodies. Cell membranes were stained with CellMask Deep Red for fluorescence imaging. E) The relative binding percentage of 1D6 and 5C8 to the surface‐displayed F‐trimer mutants and the wild‐type (n = 3). Data are presented as mean of three replicates. F) Difference analysis of the homology to human Ig between antibodies derived from diverse species (n = 9). The two‐tailed *P* values are computed using the unpaired t‐test. ****P < 0.0001. G) Superimposition of the variable region structures of 1D6, 5C8, and a representative IGHV4‐59 human Ig (PDB ID: 7FAB). CDRH3s are shown as ribbon diagrams beneath a transparent surface.

To elucidate the neutralization mechanisms of cnAbs, we labeled 1D6 and 5C8 with fluorescein isothiocyanate (FITC) and Alexa Fluor 647 (AF647), respectively and assessed their competitive binding on native F‐trimers. Consistent with competition and structural analyses using recombinant sF (Figure 6; Figures S2 and S10A, Supporting Information), both cnAbs bound equivalently to membrane‐displayed F without mutual interference (**Figure** **7**A,B). We subsequently examined the fusion of 293T cells co‐transfected with full‐length G and F expression plasmids in the presence of the antibodies. The presence of one cnAb nearly completely blocked cell fusion (Figure 7C), and the binding signal of the other cnAb was comparable to that of the control transfected with only the F‐expressing plasmid (Figure 7D), indicating that F‐trimers were locked in the prefusion conformation.

We created 66 single‐point mutants of full‐length F by mutating residues on the interface and two flanking residues on each side. We subsequently employed flow cytometry analysis to pinpoint critical sites for 1D6 or 5C8 binding. These two cnAbs, with non‐overlapping, conformation‐depended epitopes, served as crossover controls to rule out expression incompetence or misfolding of F. Both cnAbs maintained a relative binding proportion to the wild‐type F above 50% for most (48/66) mutants (Figure 7E), indicating that mutations at these sites did not impact the expression, display, or conformation of F. Only N51 was identified as a critical binding site for 5C8 (Figure 7E; Figure S10B,C, Supporting Information), correlating with its significant contribution to H‐bonds (Figure 5G). Due to the robust and dense interaction network near the predominant CDRH3 (Figure 5C), a single amino acid mutation, even R244, which is involved in three H‐bonds (two with Y100A^CDRH3^ and one with S100^CDRH3^), barely affected 1D6 binding (Figure 7E).

In summary, we propose that the tight dual‐protomer recognition and hollow filling mode of 1D6 and 5C8 enhances the rigidity of the F‐trimer, making it more conducive to locking F into its prefusion structure and less sensitive to viral variations.

### 2.6 Most CnAbs Used a Human IGHV4‐59‐like VH Framework to Form a Pushpin‐Shaped Paratope

We investigated whether shared sequences or structural features exist among potent cnAbs. Using the basic local alignment search tool,^[^ ^56^ ^]^ we determined the human immunoglobulin (Ig) that most closely matched the amino acid sequence of the variable region of F‐specific antibodies (Figure S11A, Supporting Information). The mean identities of VH and VL of reported murine‐derived antibodies to human Ig were 70.02% and 72.2%, respectively. In contrast, the cnAbs described here exhibited significantly higher homology, with VH and VL sequence identities of 88.89% and 88.7%, respectively (Figure 7F). Although the coding sequences mapped to diverse macaque germline genes (Figure S3D, Supporting Information), all eight IGHV4 cnAbs matched the same human germline gene, IGH4‐59 (UniPro ID: P01825; GenBank ID: QIH97819.1) (Figure S11A, Supporting Information). The differences in the VH of all IGHV4‐59‐like antibodies were primarily in CDRH3, while the remaining regions, including CDRH1 and CDRH2, were highly similar to those of human IGH4‐59 (Figure S11B, Supporting Information). Conversely, the VL of the nine cnAbs exhibited significant variability (Figure S11C, Supporting Information).

To eliminate interference caused by individual gene defects or use biases, we performed single‐cell sequencing on 8223 antigen‐specific memory B cells from the same individual. We obtained 4505 IGHV, 2425 IGKV, and 2216 IGLV genes (Figure S12A, Supporting Information). Allelic diversity was observed within the animal, with heavy, kappa, and lambda chains using 95, 51, and 46 alleles, respectively (Figure S12B, Supporting Information). For IGHV, all seven families were used, and the proportion of alleles among the different families was similar to previously reported data.^[^ ^57^ ^]^ We then analyzed the amino acid sequences of the IGHV4 family members using the Abalign tool^[^ ^58^ ^]^ and found highly diverse clonotype usage among VHs (Figure S12C, Supporting Information). In total, 1982 macaca‐derived VHs were assigned to 37 alleles belonging to 9 human IGHV4 subfamilies (Figure S12D,E, Supporting Information). Notably, more than half (51.1%) of the sequences belonged to families other than IGHV4, and only 15.3% of the IGHV4 genes were mapped to human IGHV4‐59 (Figure S12A,E, Supporting Information). However, the potent cnAbs demonstrated a clear preference (8/9) for the IGHV4‐59‐like skeleton (Figure S11A, Supporting Information), suggesting the potential benefit of its structure in functional activity. Unlike the representative human antibody (PDB ID: 7FAB) using IGHV4‐59, the long CDRH3 of 1D6 and 5C8 protruded markedly from the slightly cambered surface formed by the other five CDRs (Figure 7G). We speculated that all IGHV4 cnAbs might have a pushpin‐shaped structure similar to 1D6 and 5C8, wherein the IGHV4‐59‐like skeleton is conducive to the formation of a complementary concave surface to match the F‐trimer, and the long CDRH3 is easily inserted into the hollows formed by the adjacent protomers.

### 2.7 Prefusion Conformation Locking (PCL) Sites of F Are Important Targets for Structure Stabilization or CnAbs Recognition

**Figure 8:**
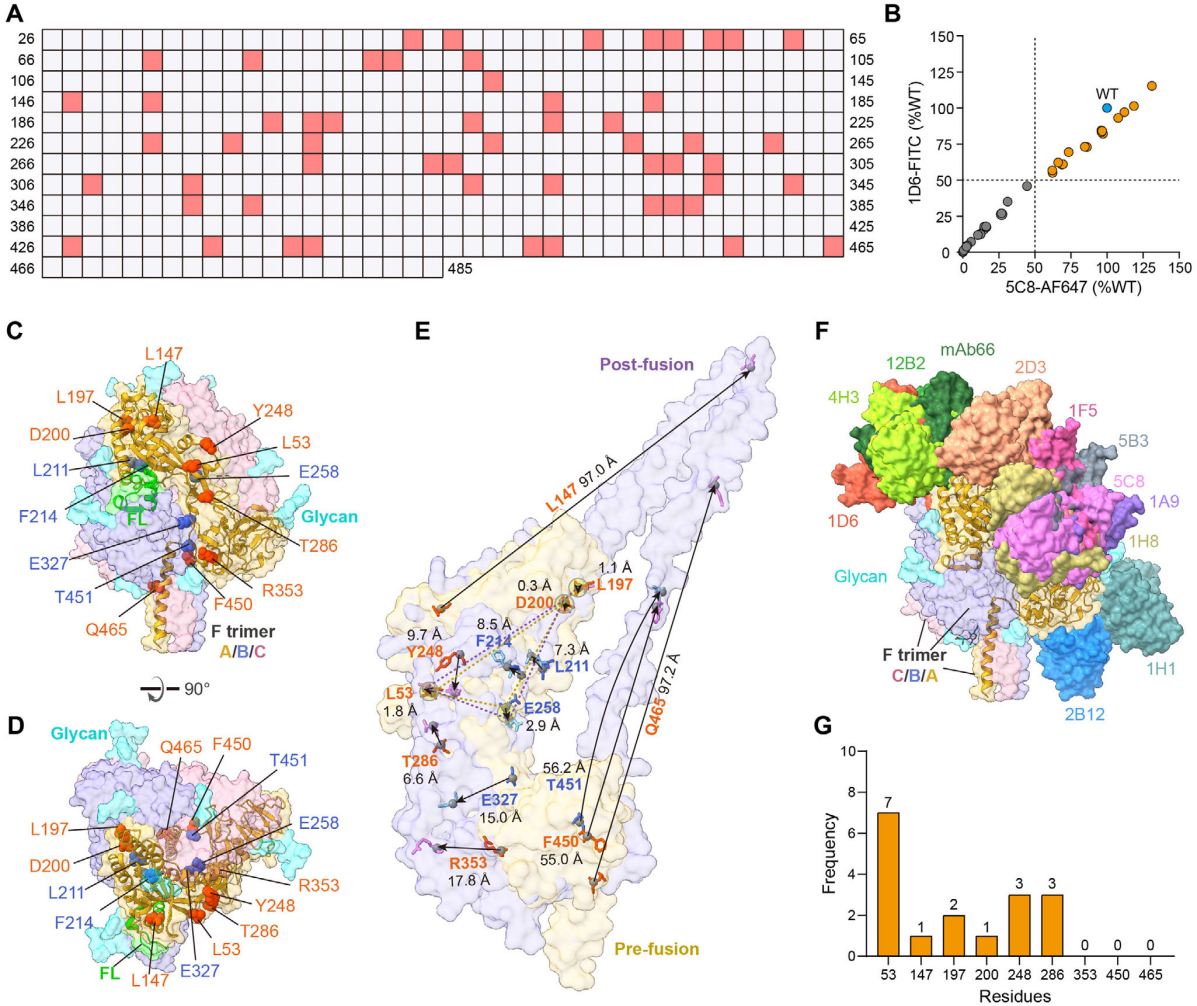
Prefusion conformation locking sites of F are important targets for structure stabilization or cnAbs recognition. A) Identification of fusion incompetent sites spanning the entire extracellular domain of NiV F. Full‐length of F wild‐type or mutants are co‐transfected with G, and the sites that suppress syncytium formation over 75% in two repeated experiments are identified as fusion‐impaired sites (pink squares). B) Binding ability of 1D6 and 5C8 to surface‐displayed F‐trimer mutants and the wild‐type (n = 3). Data are presented as mean of three replicates. C,D) Mapping of 14 PCL sites on a protomer of the NiV F‐timer. Protomers of F‐trimer and glycans are shown in surface representation. The representative protomer is colored in goldenrod and shown as ribbon diagrams beneath a transparent surface. The PCL residues are shown in sphere representation. Nine PCL sites on the surface are colored orange red, and five sites inside are colored royal blue. E) The superposition of the protomer of F pre and postfusion based on four PCL sites with fixed relative positions. The pre and postfusion conformations are shown in goldenrod and slate blue, respectively, as transparent surface representations. Nine surface and five inside PCL sites in pre and postfusion conformations are shown in orange, red, royal blue, hot pink, and cyan, respectively, as stick representations. The black line with an arrow indicates the position change between the Cα atom (shown in gray as sphere representation) of the same residue before and after fusion, and the distance is also indicated. The four PCL sites with relatively fixed positions in the pre and postfusion structure, L53, L197, D200, and E258, are connected by dotted lines in goldenrod and slate blue, respectively. F) Footprints of neutralizing antibodies on a protomer of the F‐trimer. The color of each molecule matches its label. G) Usage frequencies of PCL‐related sites in epitopes of known cross‐neutralizing antibodies.

Although the prefusion structures of HNV F have been determined, the critical amino acids for fusion remain largely unknown. To identify the essential sites for conformational changes in F, we constructed 460 full‐length NiV_BD_ F mutants spanning the entire extracellular domain (26–485 aa) via alanine scanning mutagenesis. When cotransfected with the G protein, 56 of the 460 mutants effectively abolished syncytium formation (**Figure** **8**A). These fusion‐incompetent sites distribute across the F protein domains without enrichment in any specific region, with 14 in DI, 9 in DII, 31 in DIII, and 2 in HR. Given the substantial impairment in the binding of 1D6 and 5C8 simultaneously, 42 mutants were deemed deleterious to expression or conformation (Figure 8B). The remaining 14 mutants almost entirely inhibited syncytium formation but had minimal or no effect on 1D6 and 5C8 binding (Figure 8B; Figure S13A, Supporting Information), indicating that they may block fusion by locking F in its prefusion conformation. When mapped onto the F‐trimer structure, five PCL sites were located internally, and nine were scattered across the surface (Figure 8C,D). To measure the potential impact of the PCL sites on the conformational change of F, we generated a model of the postfusion conformation of NiV F with the reference structure of LayV F (PDB ID: 8FEL). The root mean square deviation between the model and the reference LayV F or an incomplete postfusion NiV F (PDB ID: 8DMJ) was 0.16 (353 pruned atom pairs) and 1.34 Å (177 pruned atom pairs), respectively (Figure S13B,C, Supporting Information). Despite the dramatic changes in F before and after fusion, the relative positions of the four PCL sites (L53, L197, D200, and E258) changed minimally (Figure 8E). When superimposed using the four PCL sites as coordinates, the remaining 10 PCL sites resulted in shifts of at least 6.6 Å (Figure 8E; Figure S13D, Supporting Information). Given the dynamic nature of actual conformational changes, the four PCL sites with relatively fixed positions may also generate significant displacements during the intermediate fusion process.

We superimposed the available HNVs F antibodies from the PDB database onto the NiV F‐trimer and found that the antibody footprints aligned well with the locations of nine surface PCL sites (Figure 8F). We analyzed the frequency of nine surface PCL‐related sites, defined as the PCL site and one flanking residue on each side, in the epitopes of 13 HNVs F‐specific antibodies. Interestingly, all known HNV cross‐neutralizing antibodies used at least one PCL‐related site, while the remaining non‐neutralizing or breadth‐undetermined antibodies showed insensitivity to these sites (Figure S13E, Supporting Information). CnAbs did not engage the three PCL‐related sites at the base and stalk but used all PCL‐related sites in the middle or apex. Notably, five cnAbs, including 5C8, used L53‐related sites located at the upper edge of the central cavity at seven frequencies, and 1D6 simultaneously engaged two sets of three PCL‐related sites at the apex (Figure 8G; Figure S13E, Supporting Information). The PCL‐related sites in the epitopes of the cnAbs were not conserved between HNVs and parahenipaviruses (Figure S13E, Supporting Information), indicating that it is difficult for these cnAbs to expand their neutralizing breadth beyond the genus.^[^ ^47^ ^]^

## 3 Discussion

The high mortality rate and pandemic potential of HNVs highlight the urgency of developing intervention measures. However, no human‐usable preventive or therapeutic drugs are currently available. Herein, we identified nine cnAbs from a single non‐human primate memory B cell repertoire that recognize two distinct epitopes on the surface of the natural F‐trimer. Several antibodies demonstrated more vigorous cross‐neutralizing activity in vitro than previously reported F‐specific antibodies and were comparable to the best G‐specific antibodies reported. We found that antibodies targeting F or G differed in their resistance to HeV and NiV. The three representative cnAbs provided significant protection against NiV in hamsters. The binding features of the two strongest neutralizers from the two major epitope groups revealed two critical hollow‐like vulnerable epitopes formed by adjacent protomers of the F‐trimer. We also identified the critical structural‐functional sites in F. These findings are of great value for the design of henipavirus vaccines and therapeutics.

In recent years, several murine‐derived neutralizing antibodies targeting F‐trimers have been reported.^[^ ^36^, ^37^, ^42^, ^43^, ^45^ ^]^ Due to their relatively low identity with humans, the human anti‐mouse antibody response may limit the application of mouse antibodies.^[^ ^59^ ^]^ The antibodies described here were isolated from a rhesus macaque. They exhibited significantly higher homology with human antibodies, suggesting they may have relatively lower immunogenicity and reduced propensity for anti‐drug antibody induction. Notably, most of the potent cnAbs shared a typical sequence pattern: they acquired the human Ig IGHV4‐59‐like VH framework and long CDRH3 during VDJ recombination, providing valuable information for the selection and design of F‐targeting antibodies against HNVs or similar viruses.

The previous studies have provided detailed information about the epitopes distributed from the F‐trimer base to the apex. Antibodies that bind to more accessible epitopes on the upper half of the F‐trimer appear to more readily achieve potent neutralization.^[^ ^45^ ^]^ Additionally, antibodies positioned away from the viral membrane tend to trigger higher immune effector functions, which correlate with in vivo protection.^[^ ^60^ ^]^ Seven of the cnAbs described here bind to the apex and two to the middle. Although they compete with known antibodies, there are significant differences in the specific epitopes and binding patterns. Representative antibodies from the two epitope groups have a pushpin‐shaped structure that facilitates locking the F‐trimer in its prefusion conformation by inserting the long CDRH3 into the hollows between two adjacent F protomers. The vulnerable epitope and superior interaction pattern underpin the robust cross‐neutralizing activity of the antibodies against both pseudotyped and authentic viruses.

Close correlations between neutralization and protection have been observed in systematic analyses of the determinants of antibody‐mediated protection against viral diseases.^[^ ^60^, ^61^ ^]^ Given the significant protection provided by antibodies against NiV in stringent hamster models, it is reasonable to speculate that they would also offer good protective efficacy against more easily neutralized HeV. The antibody cocktail strategy has demonstrated ideal in vivo efficacy against multiple lineages or variants of the Ebola virus,^[^ ^62^ ^]^ Lassa virus,^[^ ^63^ ^]^ severe acute respiratory syndrome coronavirus 2,^[^ ^64^ ^]^ and Zika virus.^[^ ^65^ ^]^ Similarly, combining two G‐specific antibodies provided better protection than a single parent antibody in hamsters challenged with NIV_BD_.^[^ ^39^ ^]^ The uncertainty in predicting the pathogen for the next outbreak underscores the need for a more versatile or practical toolbox. However, a significant reduction in cross‐neutralizing activity against HeV was observed in RBD‐targeting anti‐G antibodies (1E5, m102.4, and HENV‐26), whereas antibodies targeting other epitopes (1B6, 2A4, 2E7, and HENV‐32) maintained their activity (Figure 3D). This phenomenon is consistent with the relatively weak blocking activity of these RBD‐targeting antibodies against HeV G and EB2/3 observed in a previous study^40^. Furthermore, second‐best‌ antibodies targeting other regions of the G exhibit cross‐neutralizing capabilities that are approximately an order of magnitude lower compared to those of RBD‐binding antibodies, which to some extent limit their potential for co‐use in cocktails.^[^ ^38^, ^39^, ^41^ ^]^ In contrast, several F antibodies identified in this study were comparable in neutralization breadth and potency to the most promising G RBD‐targeting antibodies. Given the potential interactions between G and F,^[^ ^48^ ^]^ antibodies targeting these two antigens may exert synergistic effects. As anticipated, the combination of RBD‐targeting antibody 1E5 and F‐binding antibody 1D6 or 5C8 demonstrated superior cross‐neutralizing efficacy compared to the parental antibodies. Thus, a hetero‐combination of antibodies targeting different antigens may be an optimal strategy for achieving broad‐spectrum protection against HNVs.

Despite the lack of evidence indicating whether G or F antibodies are more important in humans naturally infected with HNVs, F‐specific antibodies exhibit predominant neutralizing activity against the respiratory syncytial virus from the same family.^[^ ^66^ ^]^ Compared with G, F possesses more conserved sequences and similar architectures among henipaviruses,^[^ ^48^ ^]^ which may facilitate the elicitation and recognition of cross‐reactive antibodies. For multiple viruses, including HNVs, F in the prefusion conformation induces a significantly higher neutralizing titer than the postfusion form.^[^ ^67^, ^68^, ^69^, ^70^, ^71^ ^]^ Nevertheless, the spontaneous conformational transition observed in LayV F and the postfusion form generated by direct expression of the extracellular domain underscores the importance of stabilizing the metastable prefusion F.^[^ ^35^, ^72^ ^]^ Coiled trimerization motifs, such as GCNt,^[^ ^73^ ^]^ T4F,^[^ ^74^ ^]^ and clamp2^[^ ^75^ ^]^ are commonly used to stabilize trimeric proteins. Similar strategies have been applied to henipavirus F proteins.^[^ ^35^, ^42^ ^]^ However, additional elements introduced into antigens may cause undesired‌ immunogenicity, as evidenced by the presence of motif/tag‐binding antibodies (Figure 1E–G). Other feasible strategies include disulfide/hydrogen bond engineering, cavity filling, proline substitution, and charge changing.^[^ ^76^ ^]^ However, selecting mutation sites is challenging and often based on computer predictions and high‐throughput screening. We mapped a preliminary network of site‐structure‐function relationships through point‐by‐point mutagenesis (Figure 8), providing valuable information for the rational design of F. We divided the sites spanning the ectodomain into three categories: i) most membrane fusion non‐obstructing, ii) 42 expression/conformation‐compromising sites, and iii) 14 PCL sites. In structural design, the second category and six PCL sites (L53, L147, L197, D200, Y248, and T286) should be avoided as the former affects the correct expression of F, while cross‐neutralizing antibodies tend to target the latter. The remaining eight PCL sites located basal to or within the F may be ideal targets for design, as they appeared to be less affected by mutation and facilitated the locking of F in the prefusion conformation.

The present study has some limitations. A key research priority was the identification of cross‐protective antibodies against HNVs. However, limited BSL‐4 laboratory resources have constrained the evaluation of the protective efficacy of cnAbs against multiple HNVs in larger animal models. Similarly, while the antibodies described here exhibit in vitro neutralizing activity comparable to or surpassing that previously reported, supportive in vivo data are needed. Moreover, further analysis of the identified critical structural and functional sites of F is required to explore their potential application in structural stabilization and vaccine design.

In summary, we characterized several promising protective cnAbs against HNVs, identified the most vulnerable epitopes on the F‐trimer to date, revealed shared sequence and structural features among the most potent cnAbs, and determined critical structure‐function sites of the F‐trimer, thereby laying the foundation for the development of henipavirus vaccines and antibody therapies.

## 4 Experimental Section

### Experimental Animals and Ethics Statement

A six‐year‐old female rhesus macaque was purchased from the Laboratory Animal Center of the Beijing Institute of Biotechnology and housed in a clean facility. Before the experiment, the rhesus macaque was fed and monitored daily for months by trained personnel following the non‐human primate husbandry management practices of the Laboratory Animal Center. Six‐week‐old female Syrian golden hamsters were purchased from the Wuhan Institute of Biological Products. All hamsters were housed under specific pathogen‐free conditions for one week before the experiment.

The rhesus macaque studies were approved by the Institutional Animal Care and Use Committee of the Laboratory Animal Centre of the Beijing Institute of Biotechnology (Approval no. IACUC‐ DWZX‐2023‐002). Hamster challenge studies were conducted at the Biosafety Level‐4 facility of the State Key Laboratory of Virology and approved by the Life Science Ethics Committee of the Wuhan Institute of Virology (Approval no. WIVA45202306). All animal procedures complied with the relevant provisions of the National Regulations for the Administration of Laboratory Animals.

### Cells and Viruses

Expi293F cells (Thermo Fisher Scientific) were grown in Expi293 Expression Medium (Thermo Fisher Scientific) in an incubator maintained at 37 °C, 85% relative humidity, 8% CO_2_, and 125 rpm. HEK293T and Vero E6 cells (ATCC) were cultivated at 37 °C and 5% CO_2_ in Dulbecco's modified Eagle's medium (DMEM) (Gibco) supplemented with 10% fetal bovine serum (FBS), 100 µg mL^−1^ streptomycin, and 100 U/mL penicillin (Gibco). The TOP10 competent *E.coli* cells (TIANGEN) used to transform and extract plasmids were grown in Luria‐Bertani medium at 37 °C in a 220 rpm shaker.

The pseudotyped rHIV‐NiV_BD_, ‐NiV_MY_, ‐HeV, and ‐HeV‐g2 were packaged and used under biosafety level‐2 conditions at the Laboratory of Advanced Biotechnology, Beijing Institute of Biotechnology. Authentic NiV_MY_, NiV_BD_, and HeV were stored and used under biosafety level‐4 conditions at the State Key Laboratory of Virology, Wuhan Institute of Virology.

### Plasmids Construction

The DNA sequence used for in vitro transcription (IVT) reaction containing the T7 promoter, 5′ Homo sapiens hemoglobin alpha‐1 (HBA1) sequence, codon‐optimized full‐length coding sequence of NiV_BD_ (GenBank: AY988601.1) or HeV (GenBank: NC_0 01906.3) F, 3′ HBA1 sequence, and poly‐A tail (A_30_‐linker‐A_70_)^[^ ^77^ ^]^ was synthesized and then cloned into the pUC57 vector (General Biol) via the cleavage sites of the *Eco*RI and *Hin*dIII restriction enzymes.

The DNA sequence containing the tPA signal peptide sequence, coding sequence of the NiV (27–487 aa), HeV (27–487 aa), MojV (26–486 aa; GenBank: NC_02 5352.1), or LayV (22–482 aa; GenBank: OM101130.1) F ectodomain,^[^ ^35^, ^78^ ^]^ trimeric GCNt motif (MKQIEDKIEEILSKIYHIENEIARIKKLIGE), short linker (GGS), and His_6_ tag was codon‐optimized, synthesized (Sangon Biotech), and cloned into the pcDNA3.1 vector for soluble F (sF) production.

The full‐length light or heavy chains of antibodies were amplified from the linear cassettes above and then assembled into the pcDNA3.4 vector using the NEBuilder HiFi DNA Assembly Master (New England Biolabs) to prepare purified antibodies.

The DNA sequence containing the variable regions of h5B3.1 (Patent no. US15951327B2),^[^ ^54^, ^55^ ^]^ 1H8,^[^ ^45^ ^]^ 4H3,^[^ ^45^ ^]^ m102.4 (Patent No. US14026142B2),^[^ ^29^ ^]^ HENV‐26 (Patent No. WO2021097024),^[^ ^38^ ^]^ or HENV‐32 (Patent No. WO2022132710),^[^ ^38^ ^]^ followed by the human IgG1 constant region was codon‐optimized, synthesized (Sangon Biotech), and then constructed into the pcDNA3.4 vector for antibody expression.

The DNA sequence of the V_H_‐CH_1_ domain for Fab fragment generation was amplified from the full‐length heavy chain and introduced a C‐terminal His_6_ tag (ending as CDKTH‐H_6_),^[^ ^79^ ^]^ followed by construction into pcDNA3.4 vector.

The full‐length coding sequences of HNVs F or G were codon‐optimized, synthesized (Sangon Biotech), and cloned into pcDNA3.1. The cytoplasmic tail‐truncated forms of HNVs F (T5F)^[^ ^80^ ^]^ used for pseudovirus packaging and the alanine scanning mutants of HNVs F used for flow cytometry and fusion analysis were constructed using the Q5 Site‐Directed Mutagenesis Kit (New England Biolabs).

### mRNA‐HNVs F‐LNP Preparation

The pUC57‐NiV_BD_/HeV F plasmid was linearized by digestion with *Hin*d III and recovered using a MiniBEST DNA Fragment Purification Kit (TaKaRa). mRNA‐HNV F was synthesized via IVT at 37 °C for 2 h using the T7 High Yield RNA Transcription Kit containing N^1^‐Me‐Pseudo UTP (Vazyme) according to the manufacturer's instructions. The IVT products were treated with DNase I (Vazyme) at 37 °C for 15 min to digest the DNA templates and purified using the VAHTS RNA Clean Beads (Vazyme). The mRNA‐HNVs F was heated at 65 °C for 5 min, placed on ice for 5 min, followed by adding the 5′ Cap 1 structure using mRNA Cap 2′‐O‐Methyltransferase (Vazyme). Post purification and quantification, lipid‐nanoparticle (LNP) formulations of mRNA‐HNV F (mRNA‐HNV F‐LNPs) were prepared as previously described.^[^ ^81^ ^]^ The mRNA‐HNV F‐LNP formulations were filtered through a 0.22 µm filter, and the encapsulation efficiency and mRNA concentration were determined using the Quant‐iT RiboGreen RNA Reagent and Kit (Invitrogen).

### HNVs sF, Antibody, and Fab Fragment Production

HNVs sF proteins were expressed in Expi293F cells using the ExpiFectamine 293 Transfection Kit (Thermo Fisher Scientific) according to the manufacturer's instructions. Expi293F cells were seeded into a 500 mL shaker flask in 100 mL of fresh medium at a final density of 3×10^6^ viable cells mL^−1^. Then, 100 µg of pcDNA3.1‐NiV_BD_/HeV sF plasmids were incubated with 270 µL of transfection reagent at 25 °C for 15 min before addition into the cells. Approximately 20 h post‐transfection, two transfection enhancers were added to the cells, which were then cultured for another 96 h. The supernatants were harvested by centrifugation at 800 *× g* at 4 °C for 15 min and filtered with 0.22 µm disposable vacuum systems (Biosharp) before purification using a HisTrap Excel column (GE Healthcare) on an ÄKTA Pure 150 purification system (GE Healthcare).

Antibodies and Fabs were expressed in a 30 mL system using a procedure similar to that described above, with a corresponding reduction in the volume of all reagents. Plasmids (30 µg) were equally divided by pcDNA3.4‐heavy chain or pcDNA3.4‐V_H_‐CH_1_ and pcDNA3.4‐light chain. Antibodies were purified using a HiTrap rProteinA column (GE Healthcare), and Fabs were purified using a HisTrap Excel column (GE Healthcare).

The purified HNVs sF, antibodies, and Fabs were buffer exchanged into phosphate buffer solution (PBS) and filtered through a 0.22 µm syringe filter (PALL). Their concentration was determined, purity analyzed, and stored in aliquots at −80 °C.

### Rhesus Macaque Immunization

The rhesus macaque was initially vaccinated with a mixture of mRNA‐NiV_BD_ F‐LNP and mRNA‐HeV F‐LNP (each for 100 µg) via intramuscular (i.m.) route on day 0. Two boost immunizations using 100 µg of recombinant NiV_BD_ or HeV sF mixed with 150 µg of ODN 1826 VacciGrade (InvivoGen) and 0.25 mg of aluminum hydroxide adjuvant (InvivoGen) were employed on day 28 and 49, respectively, via the same route. The total volume of each injection was 1 mL. For serum separation, to evaluate the binding and neutralization titers, 5 mL venous blood samples were collected on days 0, 21, 28, 35, 42, 49, and 56, respectively. Anticoagulated blood samples (20 mL) were collected on day 77 for cell sorting.

### Protein Labeling

Before fluorescent labeling, 200 µg of NiV_BD_ sF or antibody was exchanged into freshly prepared 0.1 M sodium carbonate buffer (pH 9.0) using 0.5 mL centrifugal filters (Millipore) at a final concentration of ≈2 mg mL^−1^. For fluorescein isothiocyanate (FITC) labeling, the protein solution was incorporated with 5 µL FITC (Sigma Aldrich) at 1 mg mL^−1^ and incubated for 8 h at 4 °C in the dark. The reaction was terminated by adding NH_4_Cl to a final concentration of 50 mM and incubation at 4 °C for 2 h. For Alexa Fluor 647 (AF647) labeling, the AF647 dye (Thermo Fisher Scientific) was prewarmed to 25 °C and dissolved using 25 µL of PBS. The antibody solution was incorporated with 5 µL of reactive dye, and the reaction mixture was stirred for 1 h at 25 °C.

For biotin labeling, 200 µg of antibody was diluted in 100 µL of PBS and incorporated with 2.7 µL of 10 mM EZ‐Link Sulfo‐NHS‐LC‐Biotin (Thermo Fisher Scientific) reagent solution. The reaction mixture was incubated for 30 min at 25 °C.

All labeled proteins were adequately exchanged with PBS to remove excess dye or biotin and stored at 4 °C in amber microcentrifuge tubes (Axygen) post‐quantification.

### Single‐Cell Sorting

PBMCs were isolated from anticoagulated blood collected on day 77 using the monkey lymphocyte isolation solution (DAKEWE) and treated with Red Blood Cell Lysing Buffer Hybri‐Max (Sigma Aldrich) following the manufacturer's instructions. Cells were washed twice with PBS and resuspended in FPBS (PBS + 2% FBS). PBMCs were filtered through a 40 µm cell strainer (JET BlOFIL) and counted on a Cellometer K2 (Nexcelom Bioscience). NiV_BD_ sF‐specific memory B cells were sorted on an MA900 cell sorter with a 100 µm chip (SONY) using a previously described fluorescent cocktail strategy.^[^ ^41^ ^]^ For the staining per 5×10^5^ cells, the used volumes for PE mouse anti‐human IgG (BD Pharmingen, clone G18‐145), PerCP mouse anti‐human/NHP CD3 (BD Pharmingen, clone SP34‐2), PE‐Cy7 mouse anti‐human CD27 (Beckman, clone 1A4CD27), APC mouse anti‐human CD19 (Beckman, clone J3‐119), and NiV_BD_ sF‐FITC (2 mg/mL) were 15, 10, 10, 10, and 2 µL, respectively. PBMCs were incubated with a single fluorescence or cocktail away from light at 4 °C for 1 h. The cells were washed twice, and the single NiV_BD_ sF‐specific memory B cells were sorted into 96‐well PCR plates containing 20 U of RNasin ribonuclease inhibitor (Promega) and 20 µL of RNase‐free water (TransGen).

### Antibody Linear Cassette Construction and Expression

The variable genes were recovered from single cells through reverse transcription PCR (RT‐PCR) with the SuperScript III System (Invitrogen) and nested PCR with TransStart Taq (TransGen), successively, using primers previously reported.^[^ ^82^ ^]^ The VH and VL genes were cloned into linear cassettes^[^ ^83^ ^]^ by overlapping PCR to obtain the entire length of the light and heavy chains of the macaca‐human chimeric antibodies. For rapid expression of antibodies, 200 µL of HEK293T cells at 1×10^5^ cells mL^−1^ density were seeded into 96‐well plates and cultured to 80% confluency. The linear cassettes of light and heavy chains (each for 0.15 µg) were mixed with 0.6 µL TurboFect transfection reagent (Thermo Fisher Scientific) in 20 µL of Opti‐MEM medium (Gibco). The reagent/DNA mixture was incubated at 25 °C for 15 min before incorporation into each well. The culture supernatant was collected 36 h post transfection to identify positive clones.

### Enzyme‐Linked Immunosorbent Assay (ELISA)

Microplates were coated overnight at 4 °C with 100 µL of NiV_BD_ or HeV sF at 1 µg mL^−1^. The following day, the plates were washed and incubated with 100 µL of blocking buffer at 37 °C for 1 h. The plates were washed and incubated with 100 µL of serum dilutions, expression supernatant of the linear cassettes, or antibody dilutions at 37 °C for 1 h. After washing, 100 µL of the horseradish peroxidase (HRP)‐conjugated anti‐monkey or ‐human IgG antibody (Abcam) at 1:10 000 dilution was added to each well, and the plates were incubated at 37 °C for 1 h. The plates were washed and incubated with 100 µL of 3,3′,5,5′‐tetramethylbenzidine (TMB) single‐component substrate solution (Solarbio) for 6 min at 25 °C. The TMB reaction was stopped by incorporating 50 µL of the stop solution (Solarbio). The absorbance was measured at 450/630 nm on a SpectraMax ABS microplate reader (Molecular Devices).

NiV_BD_ sF was used for the competition analysis. After blocking, the plates were incubated with 50 µL of the first antibodies at 5 µg mL^−1^ (100‐fold of the biotinylated antibodies) for 30 min at 37 °C, followed by an additional 30 min incubation with 50 µL of the biotinylated antibodies at 50 ng/mL (Maximum EC_50_ concentration among binding antibodies against NiV_BD_ sF) for 30 min at 37 °C. After washing, the plates were incubated with 100 µL of streptavidin‐HRP (Abcam, 1:10 000 dilution) at 37 °C for 1 h. The remaining procedures were conducted as described above. The competition value was determined by the relative percent binding of biotinylated mAb in the presence or absence of the first mAb: < 33.3%, strong competition; 33.3 to 66.7%, intermediate competition; and > 66.7%, non‐competition.

All washing steps were done on a 405 TS washer (BioTek) with 300 µL washing buffer each time and repeated thrice. The solutions used were: coating buffer, 0.05 M carbonate buffer (pH 9.6); washing buffer, PBS containing 0.2% (v/v) Tween 20 (Solarbio); blocking buffer, PBS containing 2% (w/v) bovine serum albumin (BSA) (Sigma Aldrich); and diluting buffer, PBS containing 0.1% (w/v) BSA.

### rHIV‐HNVs Packaging and Neutralization Test

For rHIV‐HNVs packaging, 6 × 10^6^ HEK293T cells were seeded in a T75 flask and cultured to 80% confluency. The pcDNA3.1‐HNVs G (2 µg), pcDNA3.1‐HNVs T5F (2 µg), and pNL4‐3.Luc.R‐E‐ (16 µg) were co‐transfected into cells using Lipofectamine3000 transfection reagent (Invitrogen) according to the manufacturer's instructions. Six hours post‐transfection, the medium was replaced with 18 mL fresh medium. The supernatants were collected 48 h post‐transfection by centrifugation at 800×g for 10 min and then filtered through a 0.45 µm filter. The pseudovirus solution was stored in aliquots at ‐80 °C after the 50% tissue culture infectious dose (TCID_50_) was determined using the Reed–Muench method.

For the pseudovirus neutralization test, rHIV‐HNVs were diluted with medium to a titer of 2×10^4^ TCID_50_ mL^−1^. In 96‐well plates, 50 µL of serum or antibody dilutions were incubated with 50 µL of rHIV‐HNVs solution at 37 °C for 1 h. A total of 3×10^4^ HEK293T cells in 100 µL of medium were incorporated to each well and cultivated for 48 h. The medium was removed, and the cells were lysed with 50 µL of the Luciferase Cell Culture Lysis Reagent (Promega) at 350 rpm for 10 min. A volume of 20 µL of cell lysate was dispensed to 96‐well white plates (Corning). The relative luciferase unit (RLU) was detected using the Luciferase Assay System (Promega) on a Spark multimode microplate reader (TECAN). The neutralizing percentage was calculated as (1 – RLU ratio in the presence and absence of the antibody) ×100%.

To assess the synergistic neutralizing activity, equimolar antibody combinations were incubated with rHIV‐NiV pseudovirus at 37 °C. Neutralization efficacy was determined by comparing experimental wells against parallel wells treated with an irrelevant antibody control. Quantitative synergy analysis was performed using SynergyFinder software (v3.0) with the zero‐interaction potency (ZIP) reference model.^[^ ^84^ ^]^

### Surface Plasmon Resonance (SPR)

Antibody‐antigen binding kinetics were determined using a Biacore T200 instrument (GE Healthcare) at 25 °C in HBS‐EP buffer (Cytiva). The antibody at 1 µg mL^−1^ was captured to a Sensor Chip Protein A (Cytiva) at a 10 µL min^−1^ flow rate for 120 s. The NiV_BD_ or HeV sF proteins in serial dilutions were loaded at 30 µL min^−1^ to associate for 120 s, followed by dissociation for 900 s with the HBS‐EP buffer. Flow cells were regenerated using 10 mM Glycine‐HCl pH 1.5 (Cytiva). The NiV receptor binding protein was used as a negative control. Five representative curves were subtracted from the reference and fitted to a 1:1 binding model using Biacore T200 Evaluation software version 3.2 to compute the kinetic constants.

### Plaque Reduction Neutralization Test

Approximately 2.5×10^5^ Vero E6 cells were seeded into 24‐well plates and cultured to reach monolayer. The antibody was diluted in blank 24‐well plates using DMEM containing 2.5% (v/v) FBS at a 4‐fold ratio starting from 100 µg mL^−1^ for seven concentration gradients with a final volume of 150 µL per well. A volume of 150 µL of the virus at 1200 PFU/mL was added to antibody wells and incubated at 37 °C for 1 h after mixing with gentle shaking. The medium of cell plates was removed, and the cells were added with 250 µL of the antibody‐virus mixture and incubated at 37 °C for 1 h. The mixture was removed, and 100 µL of the DMEM containing 2% (v/v) FBS, 0.8% (w/v) methylcellulose (Sigma Aldrich), and 1% (w/v) penicillin was added to each well. The plates were incubated for 4 days at 37 °C, 5% CO_2_. The plates were fixed with 0.5 mL of 10% (v/v) neutral formaldehyde solution (Sbjbio) and inactivated by immersion in 75% alcohol. The plates were rinsed with tap water, and 250 µL of 0.5% crystal violet stain solution (Acros) was added to each well for staining for 10 min. After rinsing, the plates were dried and photographed to count the number of plaques per well. The neutralizing percentage was calculated as (1 – plaque number ratio in the presence and absence of the antibody) ×100%.

### Hamsters Challenge Studies

All female hamsters were healthy, drug‐ and test‐naïve, and weighed ≈100 g. The animals were randomly assigned to experimental and control groups, with six animals in each group. All hamsters were challenged with 1000 × LD_50_ of NiV_MY_ diluted in PBS (0.2 mL) on day 0. The experimental hamsters received 10 mg kg^−1^ of antibodies one day before or after the challenge. The control animals received an equal volume (0.5 mL) of PBS. All interventions were conducted intraperitoneally. The hamsters were monitored for signs of disease for 28 days and weighed for 14 days. The moribund and surviving hamsters were humanely euthanized following IACUC‐approved guidelines.

### Single‐Cell Sequencing

Approximately 2×10^7^ PBMCs were stained as described above, and CD3^−^CD19^+^CD27^+^IgG^+^NiV_BD_ F^+^ cells were sorted into a single tube on a BD FACSAria Cell Sorter (BD Biosciences). The cells were recovered by centrifugation at 500 × *g* for 5 min and inspected using a LUNA‐FL Automated Fluorescence Cell Counter (Logo Biosystems). The rhesus monkey VDJ library was constructed from 8223 cells on a Chromium Controller (10×Genomics) using Chromium Next GEM Single Cell 5′ Kit v2 (10×Genomics) and Chromium Next GEM Chip K Single Cell Kit (10×Genomics) with previously described primes.^[^ ^85^ ^]^ The library quality was evaluated on a Qubit4.0 Fluorometer (Thermo Fisher Scientific) using Qubit 1X dsDNA HS Assay Kits (Thermo Fisher Scientific). The length of inserted fragments was examined using a Qsep400 instrument (Caliper Life Science). Raw data files were generated using the NovaSeq X Plus (Illumina).

### Sequence Analysis

SnapGene software (Dotmatics) was used for general sequence analysis and alignment. The germlines and CDRs of antibodies were analyzed using the IMGT/V‐QUEST program v3.6.3 (https://www.imgt.org/IMGT_vquest).^[^ ^86^ ^]^ The Kabat numbering of 1D6 and 5C8 VH or VL amino acids displayed in the cryo‐EM panels was annotated using abYsis v3.4.1 (http://www.abysis.org/abysis/).^[^ ^87^ ^]^ The closest human Igs to the variable regions of F‐specific antibodies were matched in the UniProt database using the basic local alignment search tool (https://www.uniprot.org/blast).^[^ ^56^ ^]^ Cell V(D)J profiling was analyzed using the Cell Ranger 7.1.0 software (10×Genomics) with *cell ranger‐vdj_Macaca_mulatta* as the reference gene. The V families, VJ combinations, clonotypes, and diversity of variable regions of antibodies were analyzed using Abalign.^[^ ^58^ ^]^

### Electron Microscopy, Image Processing, and 3D Reconstruction

1D6 or 5C8 Fab was mixed with NiV sF at a molar ratio of 3:1 and incubated on ice for 40 min. After centrifugation at 18000 × *g*, 4 °C for 5 min, 100 µL supernatant was subjected to a Superdex 6 increase 5/150 GL column (GE Healthcare) for analysis. Peak samples were diluted to 0.02 mg mL^−1^ with PBS and dropped onto glow‐discharged 400 mesh copper grids (XXBR Technology). Excess samples were removed with a filter paper. After rinsing with deionized water, the grids were stained with 2% (w/v) phosphotungstic acid (BIOISCO) for 30 s. Excess fluid was removed using filter paper. The grids were dried naturally and photographed at 300 kV, 59 000× or 75 000× magnification on a Krios G4 Cryo‐TEM (Thermo Fisher Scientific) equipped with a CETA detector. Particles from 176 (1D6‐sF) and 272 (5C8‐sF) micrographs were used for 2D classification using cisTEM^[^ ^88^ ^]^ and cryosmart (Shuimu BioSciences) software to evaluate the Ab‐Ag complex quality.

An aliquot of 4 µL Ab‐Ag complex at 0.5 (1D6‐sF) or 0.25 mg/mL (5C8‐sF) was applied onto the glow‐discharged 400 mesh grids (Quantifoil Au R1.2/1.3), glow‐discharged 300 mesh grids (Quantifoil Au R1.2/1.3) supported with a thin layer of GraFuture reduced graphene oxide (RGO), or glow‐discharged 300 mesh grids (Quantifoil Au R1.2/1.3) supported with a thin layer of GraFuture graphene oxide (GO) (Shuimu BioSciences), respectively, and blotted for 3.0 s using 6 blot force. The prepared Au/RGO/GO grids were plunge‐frozen in liquid ethane using a Vitrobot Mark IV system (Thermo Fisher Scientific). The data were collected on a Krios G4 Cryo‐TEM (Thermo Fisher Scientific) equipped with a Falcon 4 detector and a Selectris X energy filter (GIF: a slit width of 20 eV). For 1D6‐sF complex, 5893 (Au), 753 (RGO), and 2098 (GO) micrographs were collected at a 165K magnification and an accumulated electron dose of 47.51 e^−^Å^−2^ s^−1^ with a pixel size of 0.74 Å. For 5C8‐sF complex, 1052 (Au) and 3166 (RGO) stacks were collected at a 96K magnification and an accumulated electron dose of 51.91 e^−^Å^−2^ s^−1^ with a 0.83 Å pixel size.

The micrographs were compressed, aligned, dose‐weighted, and summed using MotionCor2.^[^ ^89^ ^]^ The contrast transfer function (CTF) parameters were determined using CTFFIND‐4.1^[^ ^90^ ^]^ or cryoSPARC v3.2.0.^[^ ^91^ ^]^ After auto‐picking, the particles were extracted and subjected to two 2D classification rounds. In total, 1582646 1D6‐sF complex and 896634 5C8‐sF complex particles were selected and subjected to ab‐initio reconstruction, followed by heterogeneous refinement. Further homogeneous refinement and non‐uniform refinement were conducted for 1141629 (1D6‐sF) or 462582 (5C8‐sF) particles from the best 3D classes with applying C3 symmetry, which resulted in a 1.99 Å map for the 1D6‐sF complex and 2.30 Å map for the 5C8‐sF complex, respectively, based on the gold‐standard Fourier shell correlation criterion at FSC = 0.143. The local resolution was then calculated on the final density map.

The model of the complex was built by fitting a structure of the complex predicted by AlphaFold2^[^ ^92^, ^93^ ^]^ into the density map using ChimeraX,^[^ ^94^ ^]^ followed by a manual model building of the complex molecules in COOT^[^ ^95^ ^]^ and a real space refinement in PHENIX.^[^ ^96^ ^]^ The model statistics are summarized in Table S2 (Supporting Information).

### Structure Analysis

Postfusion NiV F was generated through the SWISS‐MODEL^[^ ^97^ ^]^ (https://swissmodel.expasy.org/) using the LayV F (PDB ID: 8FEL) as a reference structure. Structural superpositions, surface burial measurement, and Ab‐Ag interaction residue and H‐bond analyses were performed using ChimeraX.^[^ ^94^ ^]^ The π interactions were analyzed using Discovery Studio 4.5.0 (BIOVIA). The geometric criteria for π‐cation interactions of the atoms are defined by the following parameters: the formal charge > +0.5, the distance from the centroid of the π ring is < 5.0 Å, and the angle to the plane of the ring < 40°. The geometric criteria for π‐Alkyl interactions are the centroids of a π ring and an alkyl group < 5.5 Å with at least one pair of atoms within 4.5 Å.

### Flow Cytometry Analysis

To determine the ability of the antibodies to recognize the surface‐displayed F, 6 × 10^6^ HEK 293T cells were seeded into a T75 flask and cultured to 80% confluency. Fifteen micrograms of pcDNA3.1‐HNV T5F were transfected into the cells using Lipofectamine3000 transfection reagent (Invitrogen). Six hours post‐transfection, the medium was replaced with 18 mL fresh medium, followed by additional cultivation for 36 h. The medium was removed, and the cells were harvested by digestion with 5 mL of PBS containing 0.02% (w/v) ethylene diamine tetraacetic acid. The cells were washed twice using PBS by centrifugation at 500 *× g*, 4 °C for 6 min, and resuspended in FPBS. After being filtered through a 40 µm strainer, 5 × 10^5^ cells in 100 µL FPBS were assigned to each tube and incubated with 2 µg mL^−1^ antibody for 1 h at 25 °C. The cells were washed, resuspended in 100 µL FPBS, and then incubated with 10 µL of PE‐conjugated mouse anti‐human IgG for 1 h. After a final wash, the cells were resuspended and analyzed using a FACSCanto II flow cytometer (BD Biosciences). A total of 20000 events were recorded for each tube. An irrelevant isotype, 2G1, was used as the control.

To analyze the critical sites for antibody binding or the F structure, 0.5 µg of pcDNA3.1‐NiV_BD_ T5F wild‐type or mutant was transfected into HEK293T cells maintained in 24‐well plates. Cells were harvested and washed as described above and then detected with 2 µg mL^−1^ 1D6‐FITC and 5C8‐AF647.

To investigate the neutralizing mechanisms of antibodies, HEK293T cells were maintained in 24‐well plates and co‐transfected with full‐length NiV_BD_ F and G expression plasmids (0.5 µg each). The cells were incubated with 2 µg mL^−1^ of primary antibody and cultured for 36 h. Subsequently, the cells were harvested and detected using a fluorescence‐labeled non‐competing antibody.

### Cell Fusion Assay

To explore the neutralizing mechanisms of antibodies, HEK293T cells were co‐transfected with full‐length NiV_BD_ F and G expression plasmids. The cells were incubated with 2 µg mL^−1^ of 5C8 or an irrelevant antibody and cultured for 36 h. Experimental wells were incubated with 2 µg mL^−1^ of 1D6‐FITC at 25 °C for 30 min. Cells were carefully washed with PBS and subsequently incubated with 300 µL of CellMask Deep Red plasma membrane stain (Molecular Probes) at 25 °C for 10 min. Images were captured using an Operetta CLS High Content Analysis System (PerkinElmer) equipped with a 10× air objective.

The fusion incompetent residues were screened in 96‐well plates. HEK293T cells were transfected with pcDNA3.1‐NiV_BD_ F wild type or mutant and pcDNA3.1‐NiV_BD_ G (each for 0.15 µg). Thirty‐six hours post transfection, cell fusion was observed under a microscope and divided into I to IV grades from high to low, corresponding to fusion rates of 75–100%, 50–75%, 25–50%, and 0–25%, respectively. The residue that appeared in the grade IV group in two independent experiments was identified as a critical site for fusion.

Critical fusion sites were verified in 24‐well plates. F and G expression plasmids were co‐transfected into 293T cells with 0.5 µg each. Thirty‐six hours post‐transfection, cells were imaged using a Cytation 1 Cell Imaging Multimode Reader (BioTek). The fusion rate was analyzed and computed using ImageJ 1.52a.

### Statistical analysis

All quantitative data were presented as mean ± standard deviation (SD). The sample size in each study is indicated in the related figure legend (n). The EC_50_ values in the binding assays and IC_50_ values in the neutralization test were determined by fitting the data to a four‐parameter curve. The Shapiro‐Wilk normality test was used to assess the normality of the data. The log‐rank (Mantel‐Cox) test was performed in the animal challenge studies. Unpaired t‐tests were executed to evaluate the differences in CDRH3 length and the identity of the V regions with human Igs. Paired t‐tests were used to compare the IC_50_ values of the antibodies against pseudotyped and live viruses. Pearson's correlation analysis was conducted to correlate antibody neutralization and binding activity. All statistical analyses were performed using GraphPad Prism (v 8.0), and significance was defined as *P < 0.05, **P < 0.01, ***P < 0.001, and ****P < 0.0001. Further details are provided in corresponding figure legends.

## Conflict of Interest

P.F., C.Y., Y.R., Z.L., J.L., X.C., T.F., G.Z., B.S., and Z.C. are inventors of the two CN patents entitled “Broadly Neutralizing Antibodies Targeting the DIII (DI & DIII) Region of Henipavirus Fusion Glycoprotein and Application Thereof” (Patent No. ZL202311710303.8/ZL202311703202.8). These two patents describe antibodies described in this manuscript and do not impose any restrictions on the publication of the data. The remaining authors declare no competing interests.

## Author Contributions

Y.R., P.F., X.Z., and T.F. contributed equally to this work. C.Y. and P.F. conceived the research; P.F. conducted structural and mechanism studies, analyzed the data, and wrote the manuscript; Y.R. and T.F. immunized the animal, prepared proteins, and characterized the mAbs; Z.L., Z.S., and B.S. constructed plasmids; X.Z. and Y.L.Y. performed LNP encapsulation of mRNA vaccines; X.C., G.Z., and J.L. executed cell sorting; Z.C. provided support with sequences analysis; S.C., X.Z., Y.F.Y., C.P., B.Z., F.L., and E.L. designed and conducted in vitro/vivo experiments against authentic viruses; all authors participated in the editing and finalization of the manuscript.

## Supporting information



Supporting Information

## Data Availability

The cryo‐EM structures of 1D6‐ or 5C8‐Fab complexed with the NiVBD sF‐trimer have been deposited in the Electron Microscopy Data Bank (EMDB) and Protein Data Bank with the accession codes EMDB‐63982, EMDB‐63983, PDB ID 9UA9, and PDB ID 9UAA. This study also used 6TYS, 7KI4, 7KI6, 7UOP, 7UP9, 7UPA, 7UPK, 7UPB, 7UPD, 6T3F, 8FEL, 8DMJ, and 7FAB from the Protein Data Bank. The data that support the findings of this study are available from the corresponding author upon reasonable request.
