# Supplementary material for: Potent Cross‐neutralizing Antibodies Reveal Vulnerabilities of Henipavirus Fusion Glycoprotein
Source: Adv Sci (Weinh). 2025 Apr 29;12(27):2501996. doi: 10.1002/advs.202501996 (PMC12279222; doi:10.1002/advs.202501996)
Supplement: Supplementary file 1 — Supporting Information [file ADVS-12-2501996-s001.docx]

Supporting Information

**Potent Cross-Neutralizing Antibodies Reveal Vulnerabilities of Henipavirus Fusion Glycoprotein**

Yi Ren, Pengfei Fan,* Xinghai Zhang, Ting Fang, Zhengshan Chen, Yanfeng Yao, Xiangyang Chi, Guanying Zhang, Xiaofan Zhao, Bingjie Sun, Fangxu Li, Zixuan Liu, Zhenwei Song, Baoyue Zhang, Cheng Peng, Entao Li, Yilong Yang, Jianmin Li, Sandra Chiu,* Changming Yu*


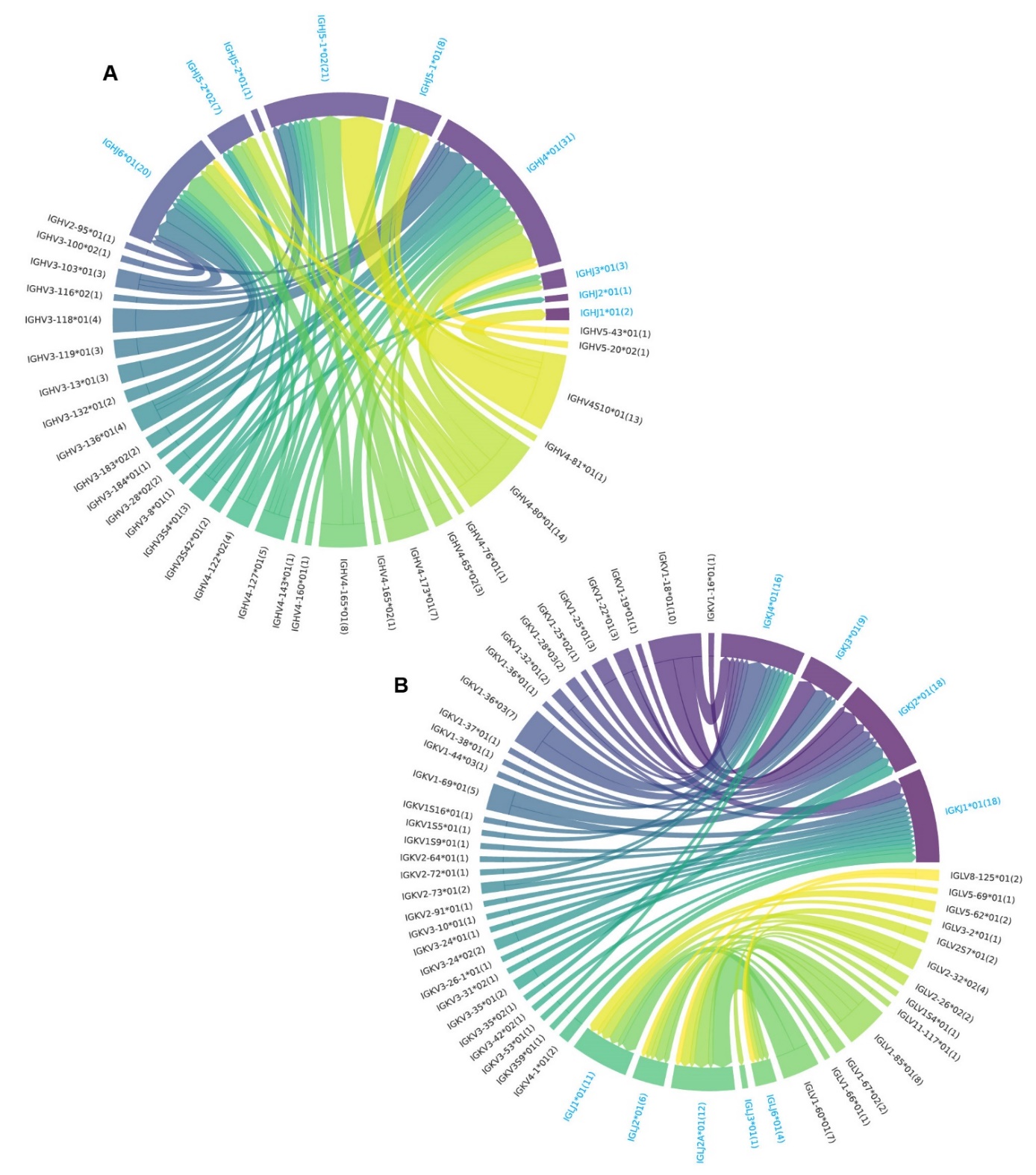


**Figure S1.** Diversity in sequences and combinations of V/J for heavy (A) or light (B) chains of positive clones.


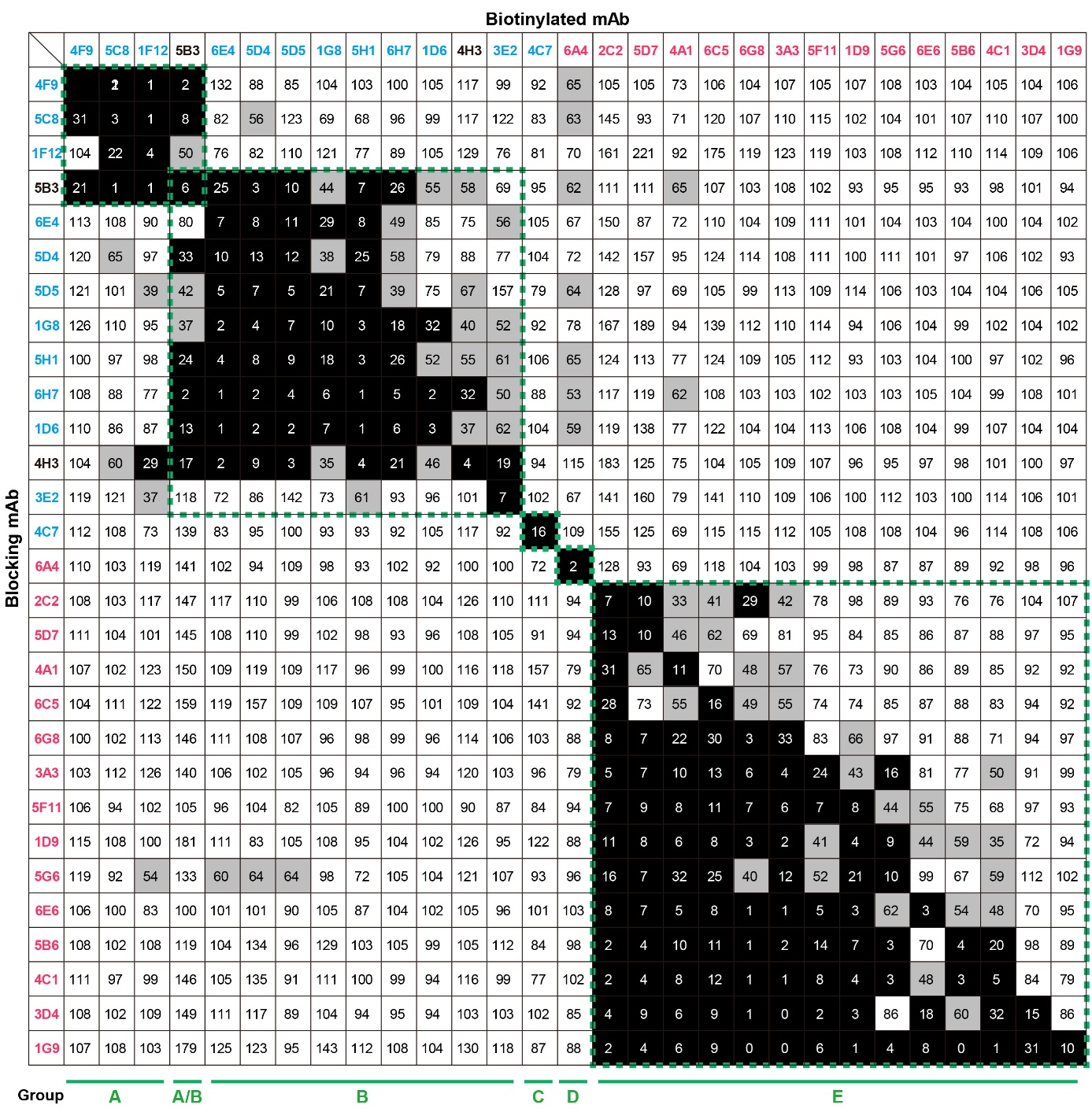


**Figure S2.** Competition-binding assay data tested using ELISA. Competitiveness is judged based on the relative percent binding of biotinylated mAb in the presence or absence of the primary mAb: < 33.3%, strong competition; 33.3 to 66.7%, intermediate competition; > 66.7%, non-competition. Inferred epitope groups are framed with blue boxes (n = 2).


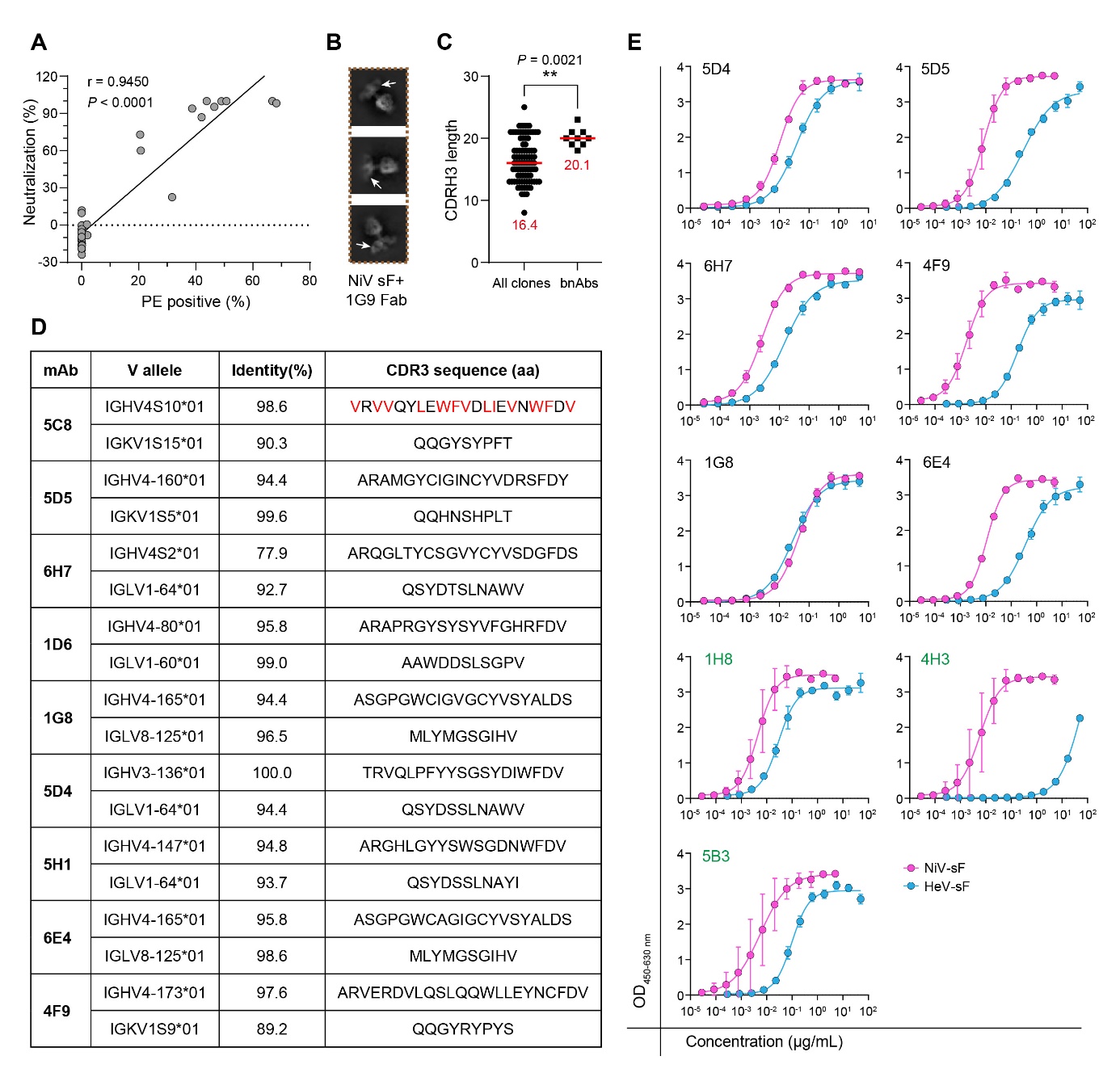


**Figure S3.** Sequence and binding profiles of cnAbs. A) Correlation between the neutralizing potency of antibodies against rHIV-NiV_BD_ and their ability to recognize the natural structure of NiV_BD_ F (n = 40). Pearson correlation coefficient and the two-tailed *P* value are computed using GraphPad Prism. *****P*<0.0001. B) Negative-stain electron microscopy of the F-trimer in complex with representative antibody 1G9 from the competing group E. C) Comparison of the mean CDRH3 length of potent cnAbs with that of all positive clones. The two-tailed *P* value is computed using the unpaired t-test. ***P*<0.01. D) Germline identity and CDRH3 sequence of neutralizing antibodies. Hydrophobic residues in the CDRH3 sequence of 5C8 are highlighted in red. E) Binding curves of cross-reactive antibodies to NiV or HeV sF determined using ELISA. The three HNVs neutralizing antibodies previously reported are marked in green (n = 3). Data are presented as mean ± SD.


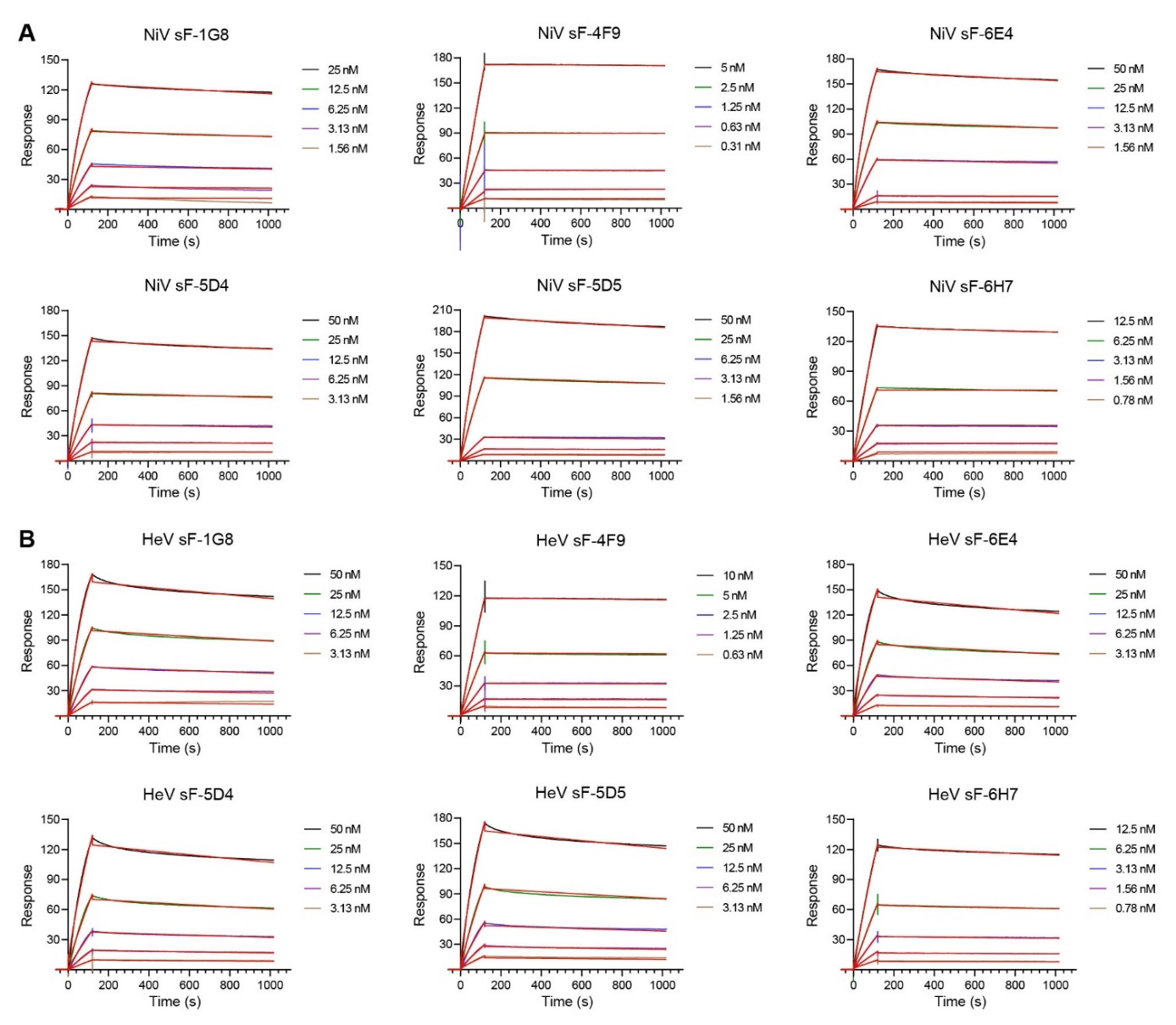


**Figure S4.** Binding kinetics of mAbs to HNVs sF determined using SPR. Five representative curves of each mAb are fitted to compute the kinetic constants.


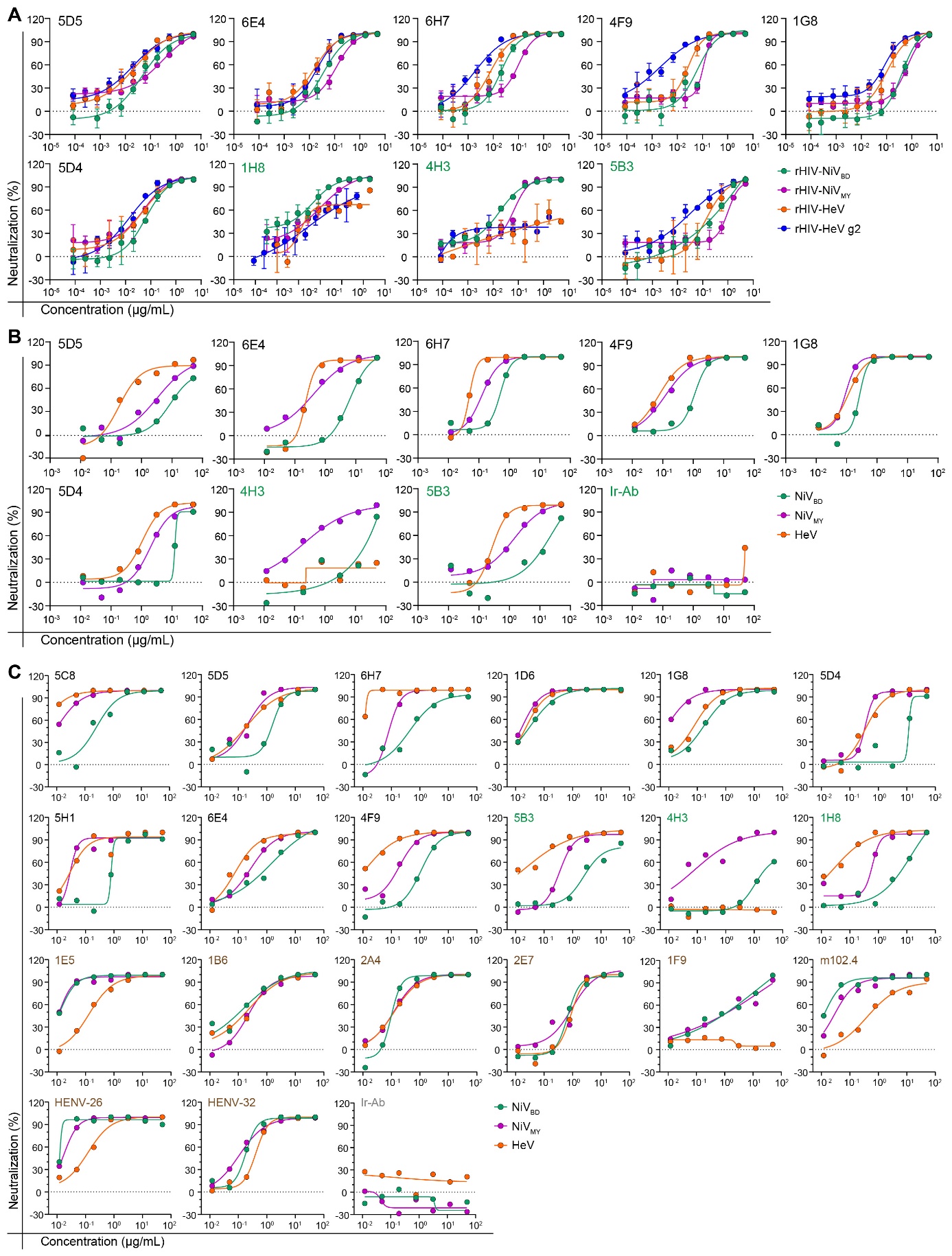


**Figure S5.** Neutralizing activity of mAbs against pseudotyped or authentic HNVs. A) Neutralization curves of mAbs against pseudotyped rHIV-NiV_BD_, -NiV_MY_, -HeV, or -HeV-g2 (n = 3). Data are presented as mean ± SD. B,C) Neutralization curves of mAbs against authentic NiV_BD_, NiV_MY_, or HeV. Data from one representative experiment are plotted (n = 2).


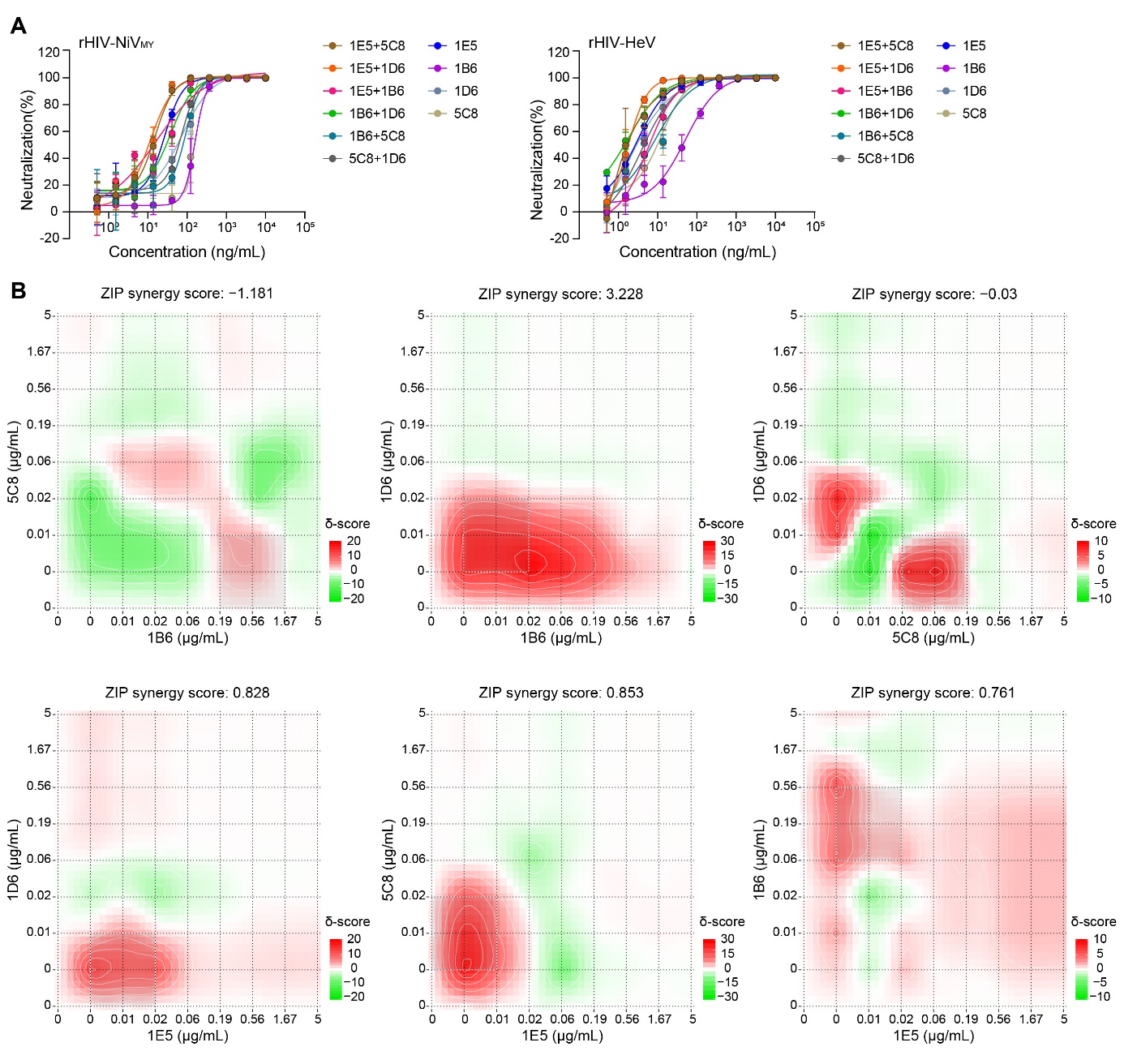


**Figure S6.** Quantitative analysis of synergistic neutralization between F- and G-specific antibodies. A) Dose-response profiles of individual mAbs versus mAb combinations against pseudotyped rHIV-NiV_MY_ or -HeV in the fixed-dose ratio test. Data are presented as mean ± SD (n = 3). All combinations maintained equimolar antibody ratios with matched total concentrations. B) Synergistic neutralization of mAb combinations against rHIV-NiV. Synergistic isobologram visualized via SynergyFinder (v3.0) and quantified using ZIP synergy scores methodology.


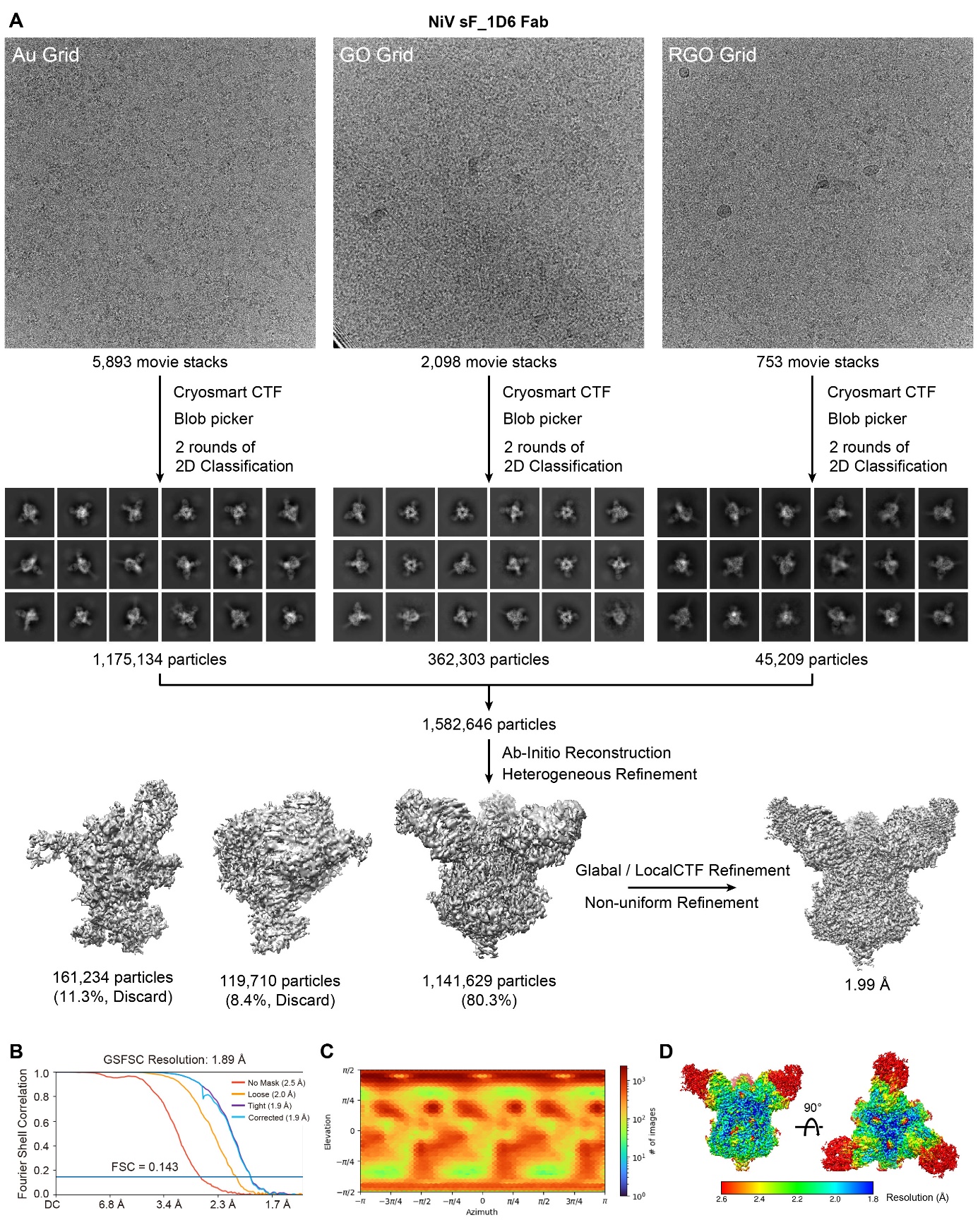


**Figure S7.** Cryo-electron microscopy data collection and processing of sF in complex with 1D6 Fab. A) Workflow of the data processing procedure. B) Gold-standard Fourier shell correlation (FSC) curves of the Ab-Ag complexes density map. C) Heat maps of particle orientation distribution for each map. D) Local resolution of the Ab-Ag complex model.


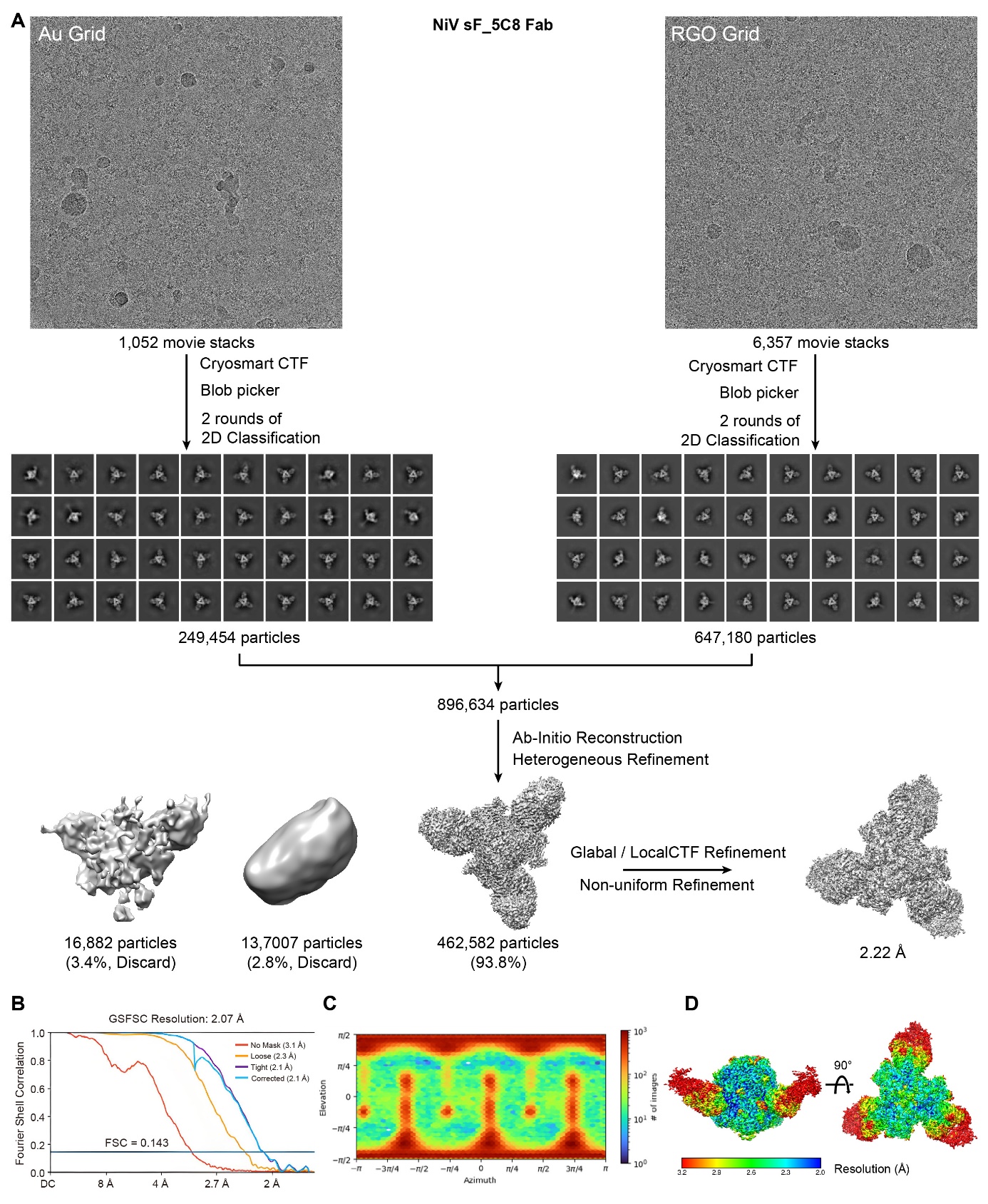


**Figure S8.** Cryo-electron microscopy data collection and processing of sF in complex with 5C8 Fab. A) Workflow of the data processing procedure. B) Gold-standard FSC curves of the Ab-Ag complexes density map. C) Heat maps of particle orientation distribution for each map. D) Local resolution of the Ab-Ag complex model.


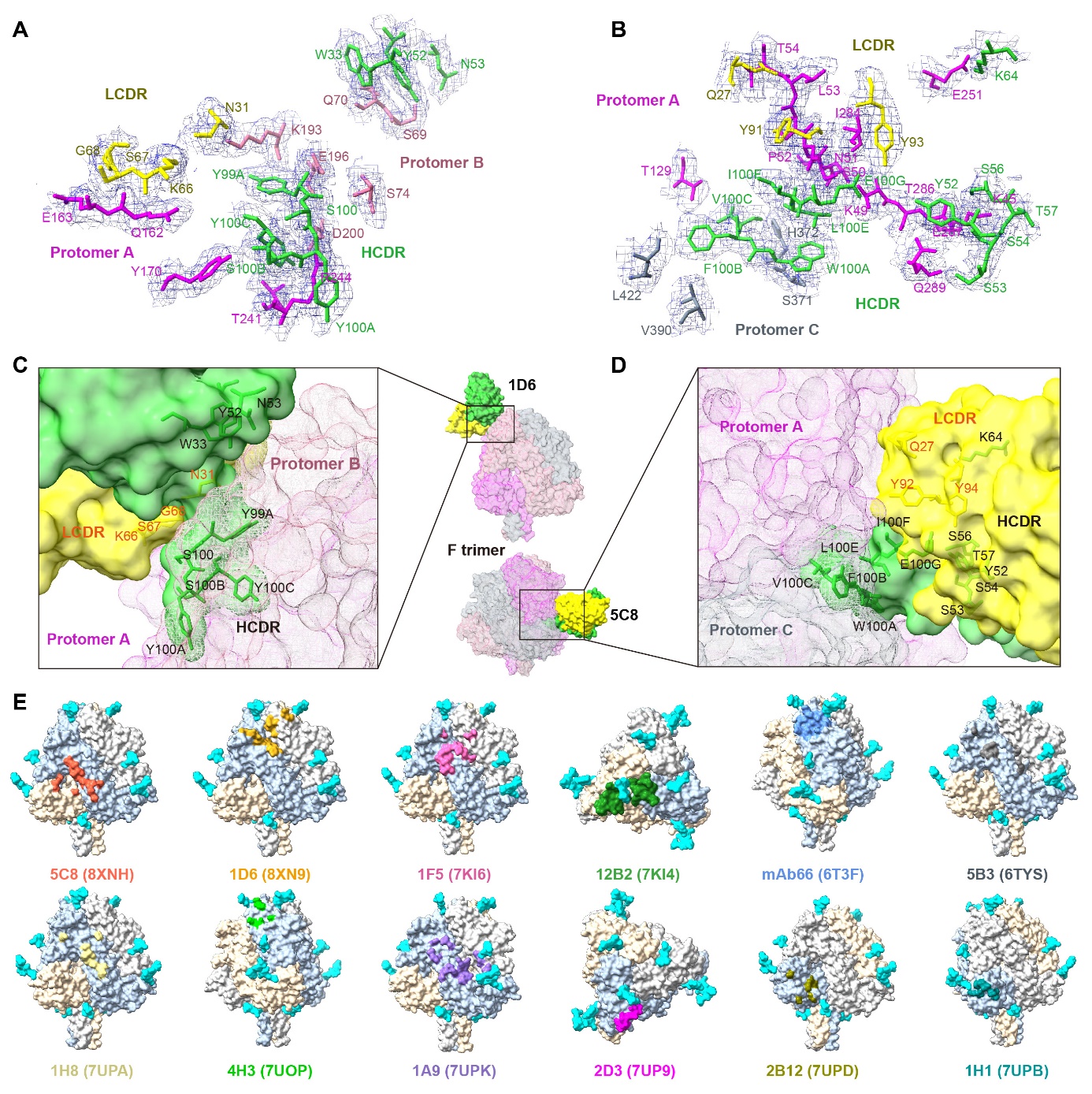


**Figure S9.** Structural details of HNVs F antibodies. A,B) Local refined density map of Ab-Ag interface with cryo-EM reconstruction shown as blue mesh and underlying fitted model in stick representation. C,D) The hollow-filling recognition mode of 1D6 (C) and 5C8 (D). Three protomers of F-trimer are colored in magenta, pale violet red, and slate gray, respectively, as transparent mesh surface representation. The VH and VL of the antibodies are shown in green and yellow, respectively, as surface representation. The key CDRH3 residues in or near hollows are depicted as stick representations. E) Footprints of F-specific antibodies on a protomer of the F-trimer. F-trimer protomers are colored in bisque, light gray, and light steel blue, respectively, and glycans are colored in cyan. Epitopes on F within 3.5 angstroms from antibodies are highlighted, and the color matches the label of each antibody.


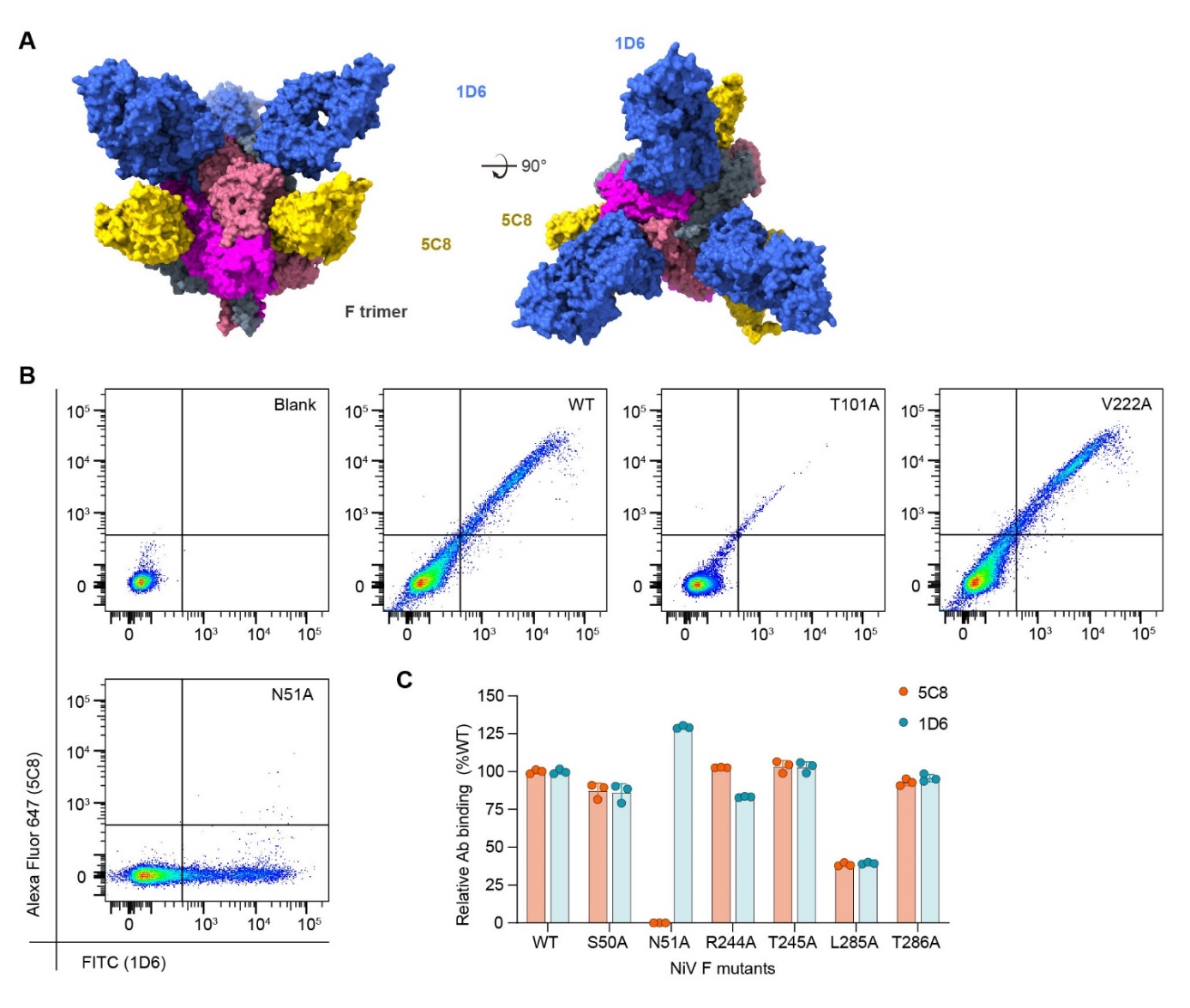


**Figure S10.** Identification of critical binding sites of 1D6 and 5C8. A) Superimposition of 1D6 and 5C8 onto NiV sF. Three protomers of F, 1D6 Fab, and 5C8 Fab are colored in magenta, pale violet red, slate gray, blue, and goldenrod, respectively, as surface representations. B) Representative flow cytometric images of 1D6 and 5C8 binding to surface-anchored T5F wild-type or mutants. C) The relative binding of antibodies to F mutants and wild type (n = 3). Data are presented as mean ± SD.


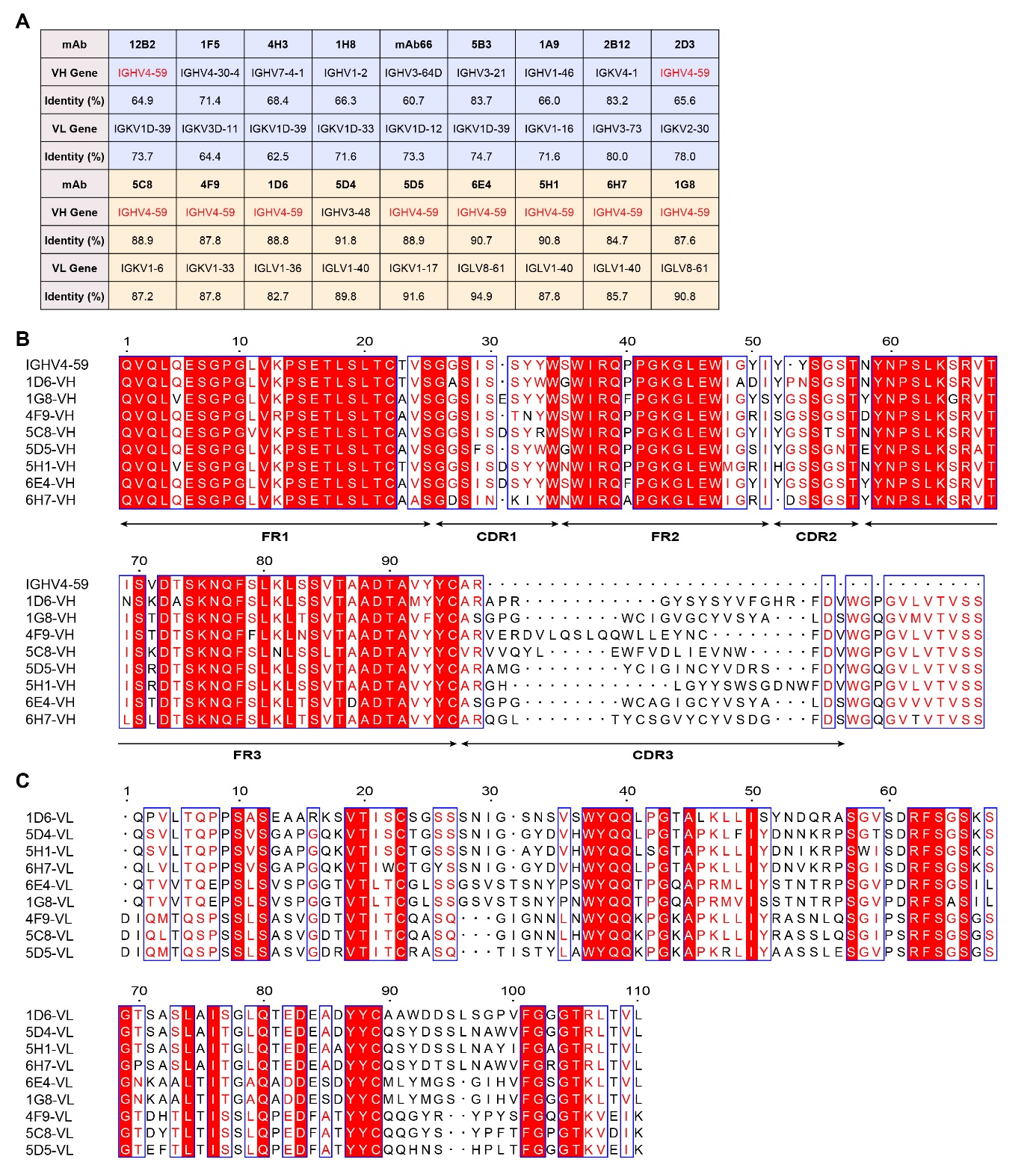


**Figure S11.** Homology analysis of amino acids of variable regions to human Ig. A) The identity of VH/VL of murine- or macaca-derived neutralizing antibodies with human Ig. The best-matched Homo sapiens Ig for each VH or VL is obtained using the Basic Local Alignment Search Tool. B) Alignment of VH sequences of eight IGHV4 macaca-derived cnAbs with human IGHV4-59. C) Alignment of VL sequences of nine macaca-derived cnAbs.


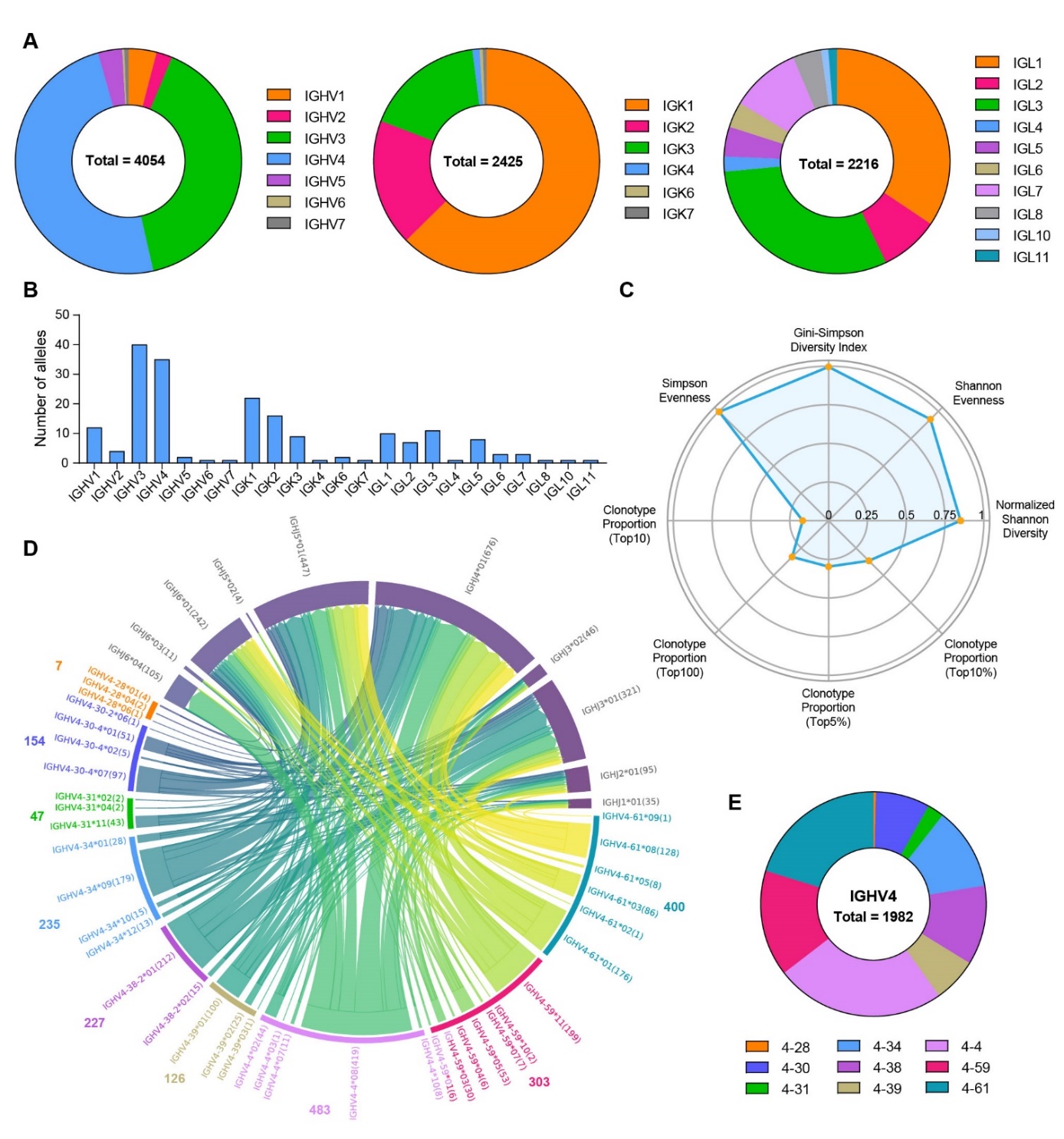


**Figure S12.** Diversity analysis of memory B-cell receptor immune repertoires of the animal. A) Distribution of heavy, kappa, and lambda gene germline families. B) The number of alleles used in diverse gene families. C) Clonotype diversity of IGHV4 sequences. D) The V-J assignment and matched human Ig alleles of IGHV4 sequences. The number in parentheses after an allele indicates the use frequency, and alleles belonging to the same subfamily are marked using the same color. The bold numbers are the number of VH genes in each subfamily. E) Distribution of human Ig subfamilies mapped by IGHV4 sequences.


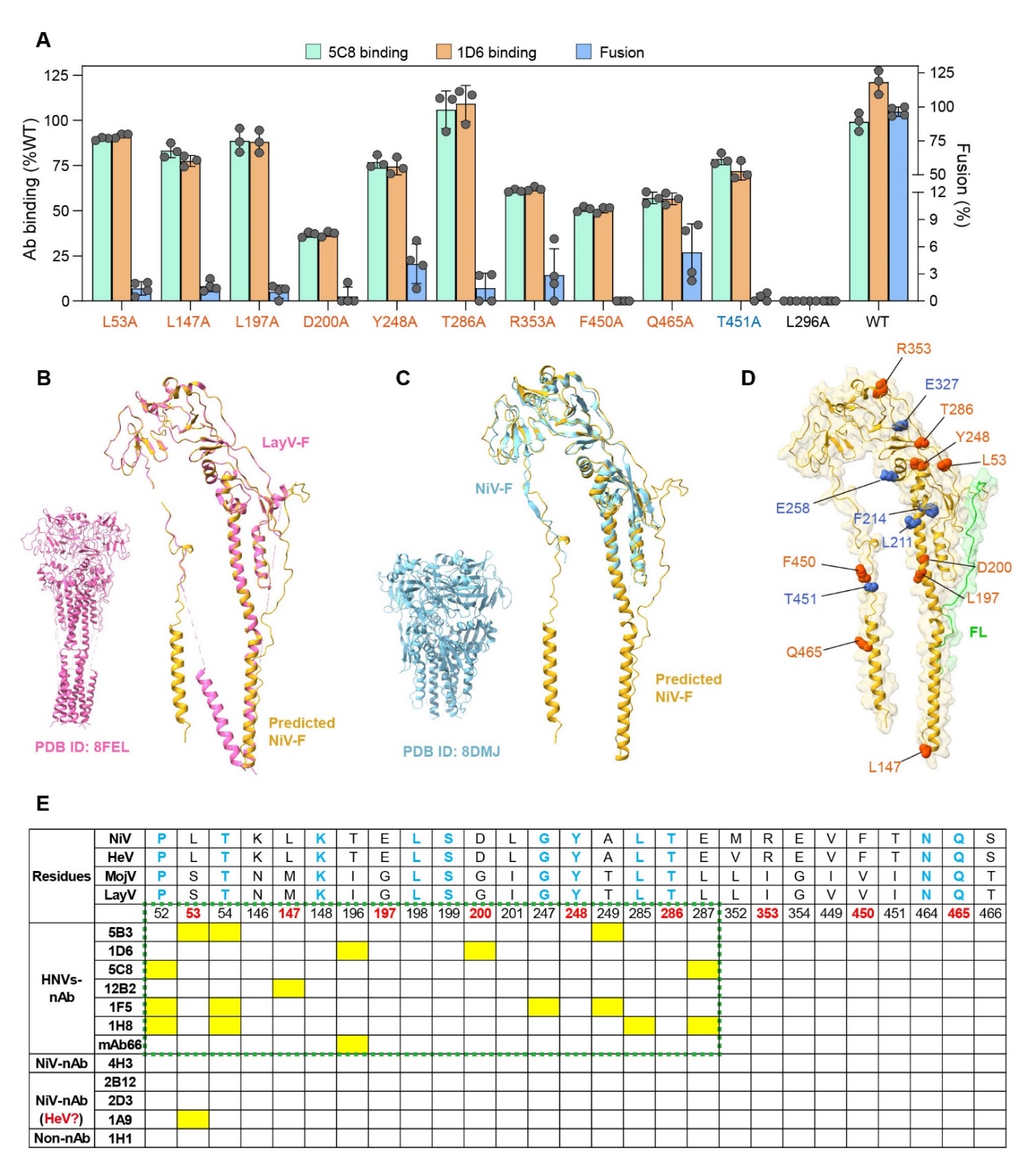


**Figure S13.** Analysis of critical structure and function sites of HNVs F. A) Effect of F mutants on antibody binding and membrane fusion (n = 3). Data are presented as mean ± SD. The left Y-axis depicts the binding percentage of antibodies to the mutant relative to the wild type, and the right Y-axis indicates the cell fusion rate when the F mutant or wild type is co-transfected with G protein. B,C) The structure superposition of the predicted postfusion NiV F with the LayV F reference model (B) or an incomplete postfusion NiV F (C) form. D) Mapping of 14 PCL sites on the postfusion NiV F protomer. The protomer is colored goldenrod and shown as ribbon diagrams beneath a transparent surface. The PCL residues are shown in sphere representation. Nine PCL sites on the surface are colored orange red, and five sites inside are colored royal blue. E) Frequency statistics of PCL-related sites in antibody epitopes.

**Table S1.** Summary of Antibody Binding Kinetics.

| mAb | Antigen | ka [1/Ms] | SE (ka) | kd [1/s] | SE (kd) | KD [M] | Chi^2^ | U-value |
| --- | --- | --- | --- | --- | --- | --- | --- | --- |
| 1D6 | HeV  sF | 5.64E+05 | 7.03E+03 | 5.15E-05 | 3.26E-07 | 9.13E-11 | 0.26 | 4.08 |
| 1G8 |  | 1.89E+05 | 1.30E+02 | 1.48E-04 | 3.35E-07 | 7.87E-10 | 2.48 | 2.41 |
| 1G9 |  | 2.27E+08 | 4.21E+06 | 4.05E-04 | 5.42E-06 | 1.79E-12 | 0.18 | 8.96 |
| 3A3 |  | 6.73E+06 | 1.38E+05 | 1.14E-04 | 9.79E-07 | 1.70E-11 | 1.69 | 6.89 |
| 3D4 |  | 2.37E+07 | 1.94E+05 | 1.07E-04 | 5.14E-07 | 4.53E-12 | 0.35 | 4.08 |
| 4F9 |  | 2.54E+05 | 5.06E+02 | 1.63E-05 | 1.62E-07 | 6.45E-11 | 0.37 | 15.14 |
| 5C8 |  | 4.75E+05 | 1.31E+03 | 1.28E-05 | 1.59E-07 | 2.70E-11 | 0.92 | 15.14 |
| 5D4 |  | 9.26E+04 | 3.72E+02 | 1.73E-04 | 4.55E-07 | 1.87E-09 | 1.32 | 1.86 |
| 5H1 |  | 1.05E+06 | 1.77E+04 | 2.79E-05 | 4.12E-07 | 2.65E-11 | 0.50 | 15.14 |
| 6E4 |  | 1.39E+05 | 1.81E+02 | 1.65E-04 | 3.14E-07 | 1.19E-09 | 1.52 | 1.86 |
| 6H7 |  | 1.08E+06 | 5.80E+03 | 1.76E-04 | 4.91E-07 | 1.64E-10 | 0.17 | 1.43 |
| 5D5 |  | 1.14E+05 | 1.33E+02 | 1.51E-04 | 3.64E-07 | 1.32E-09 | 2.92 | 2.41 |
| 1D6 | NiV  sF | 6.77E+05 | 5.83E+02 | 9.22E-05 | 3.82E-07 | 1.36E-10 | 2.21 | 4.08 |
| 1G8 |  | 6.79E+05 | 6.20E+03 | 1.08E-04 | 5.67E-07 | 1.59E-10 | 1.86 | 4.08 |
| 1G9 |  | 7.94E+05 | 1.19E+03 | 4.46E-05 | 1.16E-07 | 5.62E-11 | 0.27 | 3.14 |
| 3A3 |  | 3.56E+06 | 1.19E+05 | 1.61E-06 | 5.95E-08 | 4.53E-13 | 0.49 | 95.02 |
| 3D4 |  | 9.79E+05 | 1.70E+03 | 3.45E-05 | 2.12E-07 | 3.53E-11 | 1.08 | 8.96 |
| 4F9 |  | 2.59E+07 | 1.71E+05 | 3.26E-05 | 6.27E-07 | 1.26E-12 | 0.48 | 25.59 |
| 5C8 |  | 1.07E+08 | 1.09E+06 | 1.43E-04 | 1.80E-06 | 1.34E-12 | 0.50 | 25.59 |
| 5D4 |  | 9.49E+04 | 1.72E+02 | 7.67E-05 | 1.97E-07 | 8.07E-10 | 0.61 | 3.14 |
| 5H1 |  | 1.93E+05 | 7.73E+01 | 8.87E-05 | 1.98E-07 | 4.60E-10 | 0.95 | 1.86 |
| 6E4 |  | 1.81E+05 | 5.97E+01 | 7.53E-05 | 1.46E-07 | 4.16E-10 | 0.53 | 1.86 |
| 6H7 |  | 6.72E+07 | 2.41E+06 | 1.75E-04 | 4.17E-06 | 2.60E-12 | 1.00 | 15.14 |
| 5D5 |  | 1.09E+05 | 6.69E+01 | 7.86E-05 | 1.36E-07 | 7.19E-10 | 0.58 | 1.86 |

**Table S2.** Cryo-EM Data Collection, Refinement, and Validation Statistics.

|  | NiV sF_1D6-Fab  (EMDB-63982)  (PDB 9UA9) | NiV sF_5C8-Fab  (EMDB-63983)  (PDB 9UAA) |
| --- | --- | --- |
| **Data collection and processing** |  |  |
| Magnification | 165k | 96k |
| Voltage (kV) | 300 | 300 |
| Electron exposure (e^–^/Å^2^) | 47.51 | 51.91 |
| Defocus range (μm) | -0.4~-2.6 | -0.8~-2.6 |
| Pixel size (Å) | 0.74 | 0.83 |
| Symmetry imposed | C3 | C3 |
| Initial particle images (no.) | 1,442,575 | 493,171 |
| Final particle images (no.) | 1,141,629 | 462,582 |
| Map resolution (Å) | 1.89 | 2.07 |
| FSC threshold | 0.143 | 0.143 |
| Map resolution range (Å) | 1.7-4.0 | 1.9-6.0 |
| **Refinement** |  |  |
| Initial model used (PDB code) | AF2 predicted | AF2 predicted |
| Model resolution (Å) | 1.99 | 2.22 |
| FSC threshold | 0.5 | 0.5 |
| Model resolution range (Å) | 1.84-1.99 | 2.00-2.22 |
| Map sharpening *B* factor (Å^2^) | -57.6 | -57.5 |
| Model composition |  |  |
| Protein residues | 2031 | 2013 |
| R.m.s. deviations |  |  |
| Bond lengths (Å) | 0.002 | 0.003 |
| Bond angles (°) | 0.546 | 0.641 |
| Validation |  |  |
| Clashscore | 1.58 | 4.44 |
| Poor rotamers (%) | 0.11 | 0.00 |
| Ramachandran plot |  |  |
| Favored (%) | 98.11 | 96.38 |
| Allowed (%) | 1.89 | 3.62 |
| Disallowed (%) | 0.00 | 0.00 |
